# Engineering the unicellular alga *Phaeodactylum tricornutum* for high‐value plant triterpenoid production

**DOI:** 10.1111/pbi.12948

**Published:** 2018-06-19

**Authors:** Sarah D'Adamo, Gino Schiano di Visconte, Gavin Lowe, Joanna Szaub‐Newton, Tracey Beacham, Andrew Landels, Michael J. Allen, Andrew Spicer, Michiel Matthijs

**Affiliations:** ^1^ Eden Laboratory Algenuity Stewartby UK; ^2^ Wageningen Universiteit en Researchcentrum Bioprocess Engineering Wageningen The Netherlands; ^3^ PML: Plymouth Marine Laboratory Plymouth UK; ^4^ Rothamsted Research Harpenden UK; ^5^ Biosciences College of Life and Environmental Sciences University of Exeter Exeter UK

**Keywords:** triterpenoid biosynthesis, natural product, algal synthetic biology, diatoms, microalgae, lupeol, betulin, blue biotechnology

## Abstract

Plant triterpenoids constitute a diverse class of organic compounds that play a major role in development, plant defence and environmental interaction. Several triterpenes have demonstrated potential as pharmaceuticals. One example is betulin, which has shown promise as a pharmaceutical precursor for the treatment of certain cancers and HIV. Major challenges for triterpenoid commercialization include their low production levels and their cost‐effective purification from the complex mixtures present in their natural hosts. Therefore, attempts to produce these compounds in industrially relevant microbial systems such as bacteria and yeasts have attracted great interest. Here, we report the production of the triterpenes betulin and its precursor lupeol in the photosynthetic diatom *Phaeodactylum tricornutum*, a unicellular eukaryotic alga. This was achieved by introducing three plant enzymes in the microalga: a *Lotus japonicus* oxidosqualene cyclase and a *Medicago truncatula* cytochrome P450 along with its native reductase. The introduction of the *L. japonicus* oxidosqualene cyclase perturbed the mRNA expression levels of the native mevalonate and sterol biosynthesis pathway. The best performing strains were selected and grown in a 550‐L pilot‐scale photobioreactor facility. To our knowledge, this is the most extensive pathway engineering undertaken in a diatom and the first time that a sapogenin has been artificially produced in a microalga, demonstrating the feasibility of the photo‐bio‐production of more complex high‐value, metabolites in microalgae.

## Introduction

Plants produce a wide variety of secondary metabolites that are involved in development, defence and interaction with the environment (Moses *et al*., 2013; Sawai and Saito, 2011). Alkaloids, flavonoids and terpenoids are the major classes of plant secondary metabolites with members of all three classes already being exploited as high‐value therapeutics, flavours, fragrances and colourants (Bansal *et al*., 2016; Bourgaud *et al*., 2001; Moses *et al*., 2013; Paddon *et al*., 2013; Rouck *et al*., 2017).

Terpenoids are a structurally diverse group, which includes carotenoids, sterols and tocopherols. The number of isoprene units that are used in their synthesis determines their classification; for example, diterpenoids consist of four isoprene units and have 20 carbon atoms (C20), while triterpenoids consist of six isoprenoid units and have 30 carbon atoms (C30).

Triterpenoids constitute a wide and diverse class of plant natural products. Basic triterpenoids are termed sapogenins that often undergo chemical modifications such as oxidation, (de)methylation and the addition of nitrogen or sulphur atoms (Augustin *et al*., 2011; Kvasnica *et al*., 2015; Moses *et al*., 2013). One of the most common modifications is glycosylation, which converts sapogenin backbones into amphipathic saponins. Simple and conjugated triterpenes have a wide range of applications in the food, health and industrial biotechnology sectors (Augustin *et al*., 2011; Thimmappa *et al*., 2014).

The triterpenoid saponins derive from intermediates of the phytosterol pathway, and typical biosynthesis in plants involves a minimum of three enzyme classes: oxidosqualene cyclases (OSC), which construct the basic triterpenoid scaffolds, cytochrome P450 monooxygenases (CYP), which mediate oxidations, and uridine diphosphate‐dependent glycosyltransferases, which catalyse glycosylations (Sawai and Saito, 2011). In nature, more than 100 distinct triterpenoid scaffolds have been identified; some of the best characterized include α‐amyrin, β‐amyrin, dammarenediol and lupeol (Moses *et al*., 2015).

In this study, we focused on lupeol, betulin and betulinic acid (BA) triterpenoids. Derivatives of these compounds have shown potential for the treatment of HIV and certain cancers (Cichewicz and Kouzi, 2004; Drag *et al*., 2009; Kommera *et al*., 2011).

Although there is substantial industrial interest in triterpenoids, large‐scale extraction from their natural hosts is often costly, as these compounds are usually present in low amounts and in complex mixtures of related compounds, making their isolation difficult. Moreover, increasing the yield of target triterpenoids in the plants that naturally produce them is often unfeasible, as most medicinal plants do not have an established transformation procedure or cannot be grown on a suitable commercial scale. Additionally, the transfer of the relevant biosynthetic enzymes into other plant species is often complicated by the presence of native enzymes that can make undesirable modifications to intermediates or to the end product, as seen in *Nicotiana benthaniama* and *Oryza sativa* (Huang *et al*., 2015; Khakimov *et al*., 2015). Therefore, the investigation of biotechnological routes to produce these compounds in industrially relevant microbial hosts has become an active field of research with significant efforts being undertaken to isolate the key enzymes involved in these plant‐specific biosynthetic pathways. In general, eukaryotic hosts like *Saccharomyces cerevisiae* seem preferable for the production of triterpenoids as they can tap into the existing sterol biosynthesis pathway that is present in all eukaryotes but rare in prokaryotes (Wei *et al*., 2016). Additionally, cytochrome P450 enzymes generally do not function well in bacteria such as *Escherichia coli* and extensive engineering is often required to obtain high titres (Pateraki *et al*., 2015; Rouck *et al*., 2017).

Although the most popular heterologous hosts for terpenoid production are bacteria and yeasts (Asadollahi *et al*., 2008; Kirby *et al*., 2008; Leavell *et al*., 2016; Moses *et al*., 2015; Zhou *et al*., 2016), there has been increasing interest in photosynthetic microbial platforms, as they can utilize sunlight and CO_2_ to obtain energy and carbon for growth, thereby minimizing the environmental impact of production (Davies *et al*., 2015; Gimpel *et al*., 2015; Lauersen *et al*., 2016; Ruiz *et al*., 2016; Wijffels *et al*., 2013; Work *et al*., 2012, 2013). Moreover, algal biomass can be a useful source of a variety of value‐added molecules such as pigments, oils and omega 3 fatty acids, which can find application in the food, cosmetics, aquaculture and animal feeds industries.

Advances in synthetic biology have seen important breakthroughs in terpenoid engineering with some photosynthetic hosts, including cyanobacteria and moss tissue cultures, being successfully exploited for heterologous terpenoid production (Anterola *et al*., 2009; Arendt *et al*., 2016; Davies *et al*., 2014).

Most of microalgal research has been performed on green algae such as *Chlamydomonas reinhardtii* as they have been historically adopted as model systems to understand photosynthesis and aspects of physiology such as circadian clocks (Calvin, 1962; Noordally and Millar, 2015). Recently, a sesquiterpenoid (C15) was produced in this alga (Lauersen *et al*., 2016). However, successful engineering of green algae involves overcoming the poor expression of heterologous genes that have been integrated into the nuclear genome. Often these genes are prone to gene silencing mechanisms (Cerutti *et al*., 1997; Rasala *et al*., 2014). Therefore, extensive screening needs to be undertaken to weed out lines that only express the resistance marker and identify those which express the transgene to sufficient levels (Erpel *et al*., 2016; Lauersen *et al*., 2016; Shin *et al*., 2016). While *C. reinhardtii* has proven to be a good model organism for the elucidation of physiological processes, other algal systems are better suited for both the expression of heterologous pathways and for deployment in industrial processes. In recent years, the heterokont algae have become increasingly attractive for biotechnology and metabolic engineering applications because of their high lipid content and existing production for aquaculture (Hamilton *et al*., 2016; Kroth, 2007a; Nrel 1998; Pulz and Gross, 2004; Wikfors and Ohno, 2001). Two examples of brown unicellular algae that are genetically tractable and have publically available sequenced nuclear genomes include the Eustigmatophyte *Nannochloropsis oceanica* and the diatom *Phaeodactylum tricornutum* (Apt *et al*., 1996; Bowler *et al*., 2008; Vieler *et al*., 2012). The diatom, *P. tricornutum*, has already shown industrial potential as it can grow photoautotrophically and stably express functional enzymes, although molecular tools for genetic modification are limited. Commercially interesting products that have been produced in *P. tricornutum* include antibodies and biodegradable plastics (Hempel *et al*., 2011a,b). To the best of our knowledge, the only terpenoid engineering performed in this organism has been on the carotenoid pathway (Eilers *et al*., 2016).

**Figure 1:**
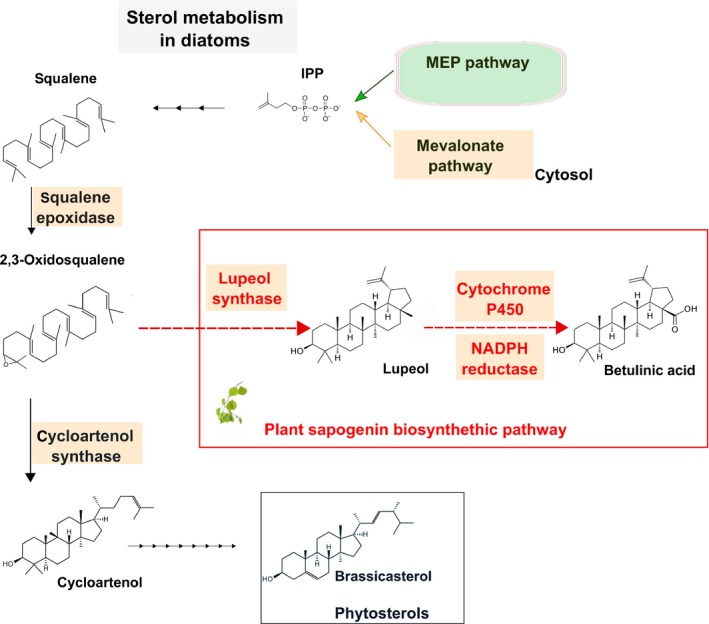
Engineering *Phaeodactylum tricornutum* by introducing a plant sapogenin pathway. Schematic representation of sterol metabolism in diatoms starting from the precursor isopentenyl pyrophosphate (IPP) and leading to brassicasterol. In the plant sapogenin pathway, the 2,3‐oxidosqualene precursor is cyclized to lupeol, by a lupeol synthase; the lupeol can be further modified to betulinic acid through a cytochrome P450 family enzyme, along with its coenzyme NADPH reductase. The plant sapogenin pathway enzymes introduced in *P. tricornutum* will, therefore, compete with the native cycloartenol synthase for the common precursor 2,3‐oxidosqualene.

Cyanobacteria and green algae form isopentenyl diphosphate (IPP) exclusively through the nonmevalonate pathway (MEP). IPP is the key precursor for sterol and other isoprenoid production. Conversely, both higher plants and diatoms such as *P. tricornutum* contain both the cytosolic mevalonate (MVA) and the chloroplastic MEP pathway. Here, we report the efficient production of two plant triterpenoids (lupeol and betulin) in *P. tricornutum* by the heterologous expression of plant enzymes: an OSC and a cytochrome P450 along with its reductase. In the plant BA biosynthetic pathway (Figure 1) a lupeol synthase OSC produces lupeol, which is subsequently oxidized at the C28 position by a CYP716A family cytochrome P450 to BA. Most of the CYP716A family enzymes also produce partial C28 oxidation products, which, in the case of lupeol oxidation, are betulin and betulin aldehyde. This is the first report of genetic engineering of a eukaryotic microalga for triterpenoid (C30) production and opens the way for photo‐bioproduction of high‐value plant secondary metabolites.

## Results and discussion

### Expression of two lupeol synthases in *P. tricornutum* results in lupeol accumulation

Triterpenes, such as sterols, are synthesized via the 30‐carbon intermediate, 2,3‐oxidosqualene, which is cyclized by members of the OSC family. These enzymes generally localize to the ER and contain a membrane‐binding domain. The genomes of the microalgae *C. reinhardtii, P. tricornutum* and the moss *Physcomitrella patens* contain a single OSC gene for tetracyclic sterol biosynthesis. Higher plants, in contrast, can contain a remarkable amount of chemical diversity derived from the single substrate 2,3‐oxidosqualene. Typically plant genomes contain from nine to sixteen *OSC* genes, for example the model plant *Arabidopsis thaliana* genome contains thirteen *OSC* gene family enzymes and can produce a diverse array of triterpene skeletons (Vincken *et al*., 2007; Xue *et al*., 2012). Among these, a lupeol synthase, At1g78970/LUP1 has been characterized (Herrera *et al*., 1998), successfully expressed and used for lupeol and BA production in yeasts (Czarnotta *et al*., 2017; Husselstein‐Muller *et al*., 2001; Lang and Lewandowski, 2016; Zhou *et al*., 2016).

The *A. thaliana* lupeol synthase (from now on designated as AtLUS) catalyses the production of lupeol and the related triterpenoid, 3β, 20‐dihydroxylupane, as a by‐product (Salmon *et al*., 2016; Segura *et al*., 2000).

To select an OSC enzyme for introduction in *P. tricornutum,* we performed a phylogenetic analysis of selected plant OSCs with known functions (Figure S2); interestingly, AtLUS clusters with beta‐amyrin synthases such as the *Artemisia annua* OSC2 and the *Catharantus roseus* beta‐amyrin synthase BAS), while all lupeol synthases (LUS) cluster separately, that is *Bupleurum lanceolate LUS*,* Lotus japonicus* OSC3, *Olea europaea* LUS. It has been suggested previously that either two branches of lupeol synthase genes have been generated during higher plant evolution or that, AtLUS represents an evolutionary transition state between lupeol synthase and other unknown triterpene synthases (Shibuya *et al*., 1999).

**Figure 2:**
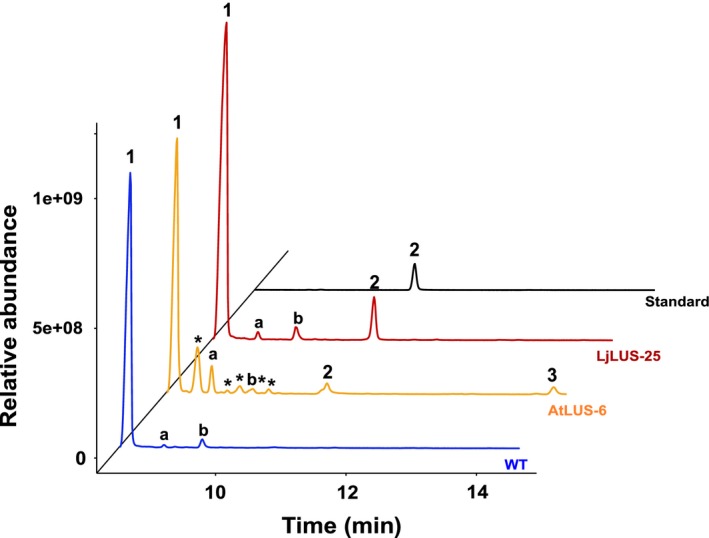
Lupeol production in *Phaeodactylum tricornutum* transformant lines. GC‐MS analysis of transformant lines AtLUS‐6 (yellow) and LjLUS‐25 (red) with the wild type (blue). Lupeol standard in black. 1. Brassicasterol (major sterol in *P. tricornutum*). 2. Lupeol; 3. 3β, 20‐dihydroxylupane by‐product of AtLUS enzyme only. Additional peaks: a. campesterol; b. unidentified diterpene molecule; *unidentified terpenoid molecules, mass spectra for these peaks with their best database hits are shown in Figure S3.

To test the feasibility of *P. tricornutum* as a lupeol production platform, we ectopically expressed two representative OSCs: AtLUS or *L. japonicus* OSC3 (from now on LjLUS). Both enzymes have been shown to be functional in *S. cerevisiae*, with AtLUS being the most prevalent in literature (Czarnotta *et al*., 2017; Sawai *et al*., 2006; Zhou *et al*., 2016). The native coding sequences of *AtLUS* and *LjLUS* were integrated into the *P. tricornutum* nuclear genome under the control of the strong fucoxanthin chlorophyll a/*c* binding protein A (*FCPA/LHCF1)* promoter, along with zeocin resistance gene marker (*ble* ^r^) (SF1, A and B). Single transformant lines grown on selective solid medium (zeocin antibiotic) were transferred to liquid medium and grown until early stationary phase. Thirty lines were subjected to GC‐MS analysis and lupeol was detected in both *LjLUS* and *AtLUS* expressing lines (Figure 2) with the identification supported by the MS electron impact spectra (Figure S3A–R) Based on the initial GC‐MS screening, we selected those lines with the highest relative production of lupeol (Table S1). On average, we were able to detect lupeol in 30% of the screened lines for each construct. This established that the enzymes are functionally expressed and that *P. tricornutum* lines expressing heterologous genes can be identified using a relatively easy and low‐throughput screening method. This compares favourably with the *C. reinhardtii* platform used for sesquiterpene patchoulol (C15) production*,* where more intricate vector design and screening methods need to be employed to select positive lines, including the preparation of a construct harbouring three copies of the transgene, one of which was fused with a reporter gene (YFP) to allow colonies to be screened for fluorescence on primary transformant plates (Lauersen *et al*., 2016).

In the transformant lines expressing AtLUS*,* we were able to identify lupeol and the 3β, 20‐dihydroxylupane by‐product, often produced at similar titres, but also dependent on culturing conditions, while LjLUS expressing lines produced just lupeol (Figure 2). We, therefore, focused our attention on line LjLUS‐25. To the best of our knowledge, this is the first successful demonstration of pentacyclic triterpenoid production in an engineered microalga.

Interestingly, no canonical squalene epoxidase enzyme has been identified in *P. tricornutum* based upon sequence or structural similarity (Fabris *et al*., 2014), although its product, the 2–3 oxidosqualene, was predicted to be present as the substrate of the well‐annotated cycloartenol synthase (Figure 1). This result affirms the presence of 2–3 oxidosqualene in *P. tricornutum*, as the introduced LUS require this precursor to synthesize lupeol.

### High lupeol productivity correlates with high *LjLUS* expression but is independent on the number of integrated expression cassettes

**Figure 3:**
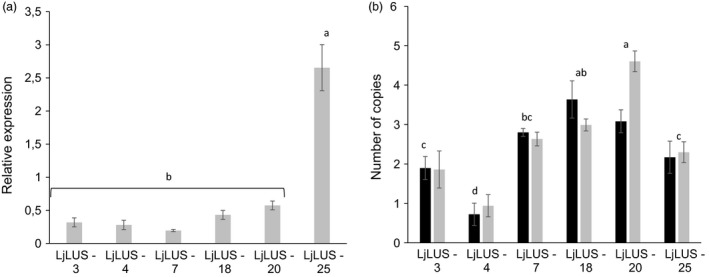
Expression and copy number of lupeol‐producing strains. (a) qPCR analysis of six lines showing expression of the *Lotus japonicus* lupeol synthase (*LjLUS*) mRNA relative to the geometric average of the *RP3a* and *UBQ* reference genes (b) Copy number of integrated *LjLUS* expression cassettes (black) and the *ble* ^*r*^ resistance marker (grey). Error bars represent the standard deviation from three biological replicates. Identical letters denote groups where means are not statistically different according to a *post hoc* Tukey test with α = 0.01.

Nuclear transformation events in *P. tricornutum* lead to random insertions of a given transgene into the nuclear genome. In order to test whether there is any correlation between higher lupeol production and relative transgene expression levels, we performed qRT–PCR on the three LjLUS lines that showed the lowest lupeol accumulation and the three lines that showed the highest lupeol accumulation. A one‐way ANOVA of LjLUS expression in the selected lines showed that there was a significant difference in expression *F*(7, 20) = 7.17, *P* < 0.01. The line with the best lupeol accumulation, namely LjLUS‐25, was significantly different as supported by a *post hoc* Tukey test (α = 0.01), while the other lines grouped together (Figure 3A).

To investigate whether mRNA expression levels are mainly determined by locus of integration or due to transgene copy number resulting from single or multiple nuclear genome integration events, the approximate transgene copy number was quantified using qRT–PCR for the three low and three high lupeol‐producing strains. Results were normalized by amplifying regions within two different wild‐type genomic loci (*RP3a Phatr3_J13566* and *UBQ Phatr3_J 28620*), for which the copy number is assumed to be two in a diploid cell. The copy number of the *LjLUS* coding sequence and the zeocin resistance marker (*ble* ^*r*^) were determined. Both transgenes were present on a single transformation vector and were found to have similar abundance: two to three transgene copies for each transformant line (Figure 3B). This is consistent with what has been previously reported for *P. tricornutum* integration events (Falciatore *et al*., 1999). An ANOVA was conducted to compare the main effects of the individual lines and the integrated transgene. This analysis confirmed that there was no difference between the number of integrated *ble* ^*r*^ and *LjLUS* copies (*P* = 0.29) but that there were significant differences between the lines (*P* < 0.001, *F*(6, 29) = 26.49). *Post hoc* comparisons were performed for the comparison of the lines and groups significantly different from each other are indicated in Figure 3B (α = 0.01). There was no correlation between mRNA expression levels and the number of integrated *LjLUS* cassettes (Pearson correlation score of −0.03, *t* = 0.07, *P* > 0.9). These results suggest that variations in relative mRNA expression and lupeol accumulation are likely due to positional (site of integration) effects rather than the absolute number of integrated transgene copies.

### Production of lupeol is growth phase dependent and reduces brassicasterol levels in the cell

**Figure 4:**
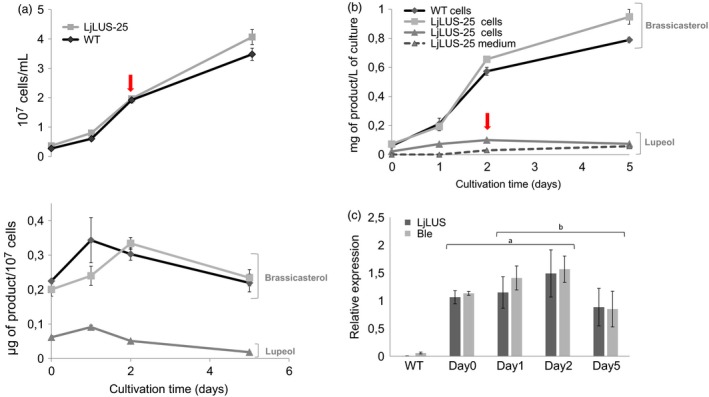
Lupeol productivity in 400‐mL batch cultures. Triplicate cultures were grown in F/2 medium in laboratory‐scale Algem^®^ photobioreactors for 5 days. (a) Cell density and productivity per cell of brassicasterol for the wild type (diamonds) and LjLUS‐25 (squares). Lupeol was only detected in the LjLUS‐25 line (triangles). (b) Brassicasterol extracted from cell pellet of WT (diamonds) and the LjLUS‐25 line (squares). Lupeol extracted from LjLUS‐25 is shown by the triangles in the cell (full line) and the medium (dashed line). No brassicasterol was detected in the medium of either the WT or LjLUS‐25. (c) Gene expression of *LjLUS* and *ble* ^*r*^ resistance marker for LjLUS‐25 transformant line (WT as negative control) monitored during culturing time and relative to the reference genes *RP3a* and *UBQ*. Red arrows indicate the day when maximum lupeol productivity is observed corresponding to mid‐exponential growth phase as described in the text. All error bars represent the standard deviation from three biological replicates. Identical letters denote groups where means are not statistically different according to a *post hoc* Tukey test with α = 0.01.

In order to monitor the lupeol productivity during the different growth phases, we performed experiments in an Algem^®^ labscale photobioreactor, collecting and analysing the biomass at days 0, 1, 2 and 5 of growth. Cells reached maximum productivity of both brassicasterol (the main sterol) and lupeol during mid‐exponential growth phase (as indicated by the arrows in Figure 4), suggesting that cells are actively engaged in sterol metabolism at the early stage of their growth. We could also observe a reduction in brassicasterol per cells in the LjLUS‐25 line when compared to WT, consistent with the amount of lupeol produced per cell (Figure 4a). The maximum yield of lupeol in the cell was reached after 2 days of growth.

No brassicasterol was observed in either the LjLUS or WT media, suggesting cellular lysis at this stage was minimal. It appears that while native sterols such as brassicasterol are retained within the cell, lupeol is able to actively or passively translocate into the medium (Figure 4b).

We also tracked gene expression of *LjLUS* and the zeocin resistance confirming protein *ble* ^*r*^ for 5 days during which the cells transitioned from exponential to stationary phase. Both transcripts showed maximum expression levels during mid‐exponential growth phase, in line with the lupeol productivity obtained (Figure 4c). An ANOVA showed that the difference in time was significant (*F*(3) = 6.32, *P* = 0.005) but there was no significant difference between the expression of *LjLUS* and *ble* ^*r*^ (*F*(1) = 0.7, *P* = 0.41). This result is also consistent with the *FCPA/LHCF1* promoter activity, which peaks during exponential growth. While the *FCPA/LHCF1* promoter is widely used, a stronger and growth phase independent promoter could potentially enhance LjLUS activity.

Strain LjLUS‐25 was selected as the best producing line. LjLUS‐25 reached a maximum lupeol yield (C30) of 0.1 mg/L over 2 days of culturing. This reported yield is similar to the 0.35 mg/L yield of lupeol achieved in *C. reinhardtii* engineered for the production of the sesquiterpene patchoulol (C15), grown in comparable conditions, over a 7‐day period (Lauersen *et al*., 2016).

For comparison: the highest yield for BA production (182 mg/L) has been achieved in a *S. cerevisiae* strain (Czarnotta *et al*., 2017). This strain is the result of multiple optimization rounds and fed‐batch cultivations. The first‐generation strain showed BA titres of 0.1 mg/L/OD (Huang *et al*., 2012), and with further genetic improvements, increased flux into the mevalonate pathway and elevated cofactor supply, BA titres reached 12.1 mg/g dry biomass (Li and Zhang, 2014; Li and Zhang, 2015). In our microalgal system, we obtained titres >0.1 mg/g dry biomass with no prior optimization, and this is higher than the initial concentrations reported for the primary yeast strains.

**Figure 5:**
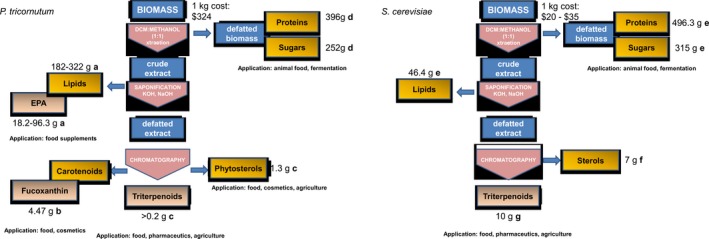
Schematic representation of proposed model for extraction procedure with a biorefinery approach of triterpenes from *Phaeodactylum tricornutum* and *Saccharomyces cerevisiae* biomass. The extraction procedure for triterpenoids from *P. tricornutum* simultaneously isolates more commercially relevant coproducts compared to the yeast *S. cerevisiae*. Cost of extraction is estimated to be assuming equal levels of extraction efficiency. Cost of biomass for *P. tricornutum* is higher (produced in Europe, Necton), but market sizes of lipids omega 3 and carotenoids (unique products in microalgae) create cost offsets. References for panel A: a: *W. Yongmanitchai and O. P. Ward Growth of and Omega‐3 Fatty Acid Production by *Phaeodactylum tricornutum* under Different Culture Conditions Appl. Env. Micro., Feb. 1991, p. 419–425 0099‐2240/91/020419‐07$02.00/0, b: Yu‐Hong Yang, Lei Du, Masashi Hosokawa, Kazuo Miyashita, Yume Kokubun, Hisayoshi Arai and Hiroyuki Taroda. Fatty Acid and Lipid Class Composition of the Microalga *Phaeodactylum tricornutum* J. Oleo Sci. 66, (4) 363–368 (2017) https://doi.org/10.5650/jos.ess16205, c: this paper, d: S. M. Tibbetts, J. E. Milley, P. Santosh and J. Lall Chemical composition and nutritional properties of freshwater and marine microalgal biomass cultured in photobioreactors J Appl Phycol (2015) 27:1109–1119. References for panel B: e: International Journal of Environment, Agriculture and Biotechnology (IJEAB) Vol‐2, Issue‐2, Mar–Apr 2017 https://doi.org/10.22161/ijeab/2.2.2 ISSN: 2456–1878 Page | 558, f: M. Lamac Ka and J. S Ajbidor Ergosterol determination in *Saccharomyces cerevisiae*. Comparison of different methods. Biotech. Tech. 11, 723–725 (1997), g: E. Czarnotta, M. Dianat, M. Korf, F. Granica, J. Merz, J. Maury, S. A. Baallal Jacobsen, J. Förster, B. E. Ebert, and L. M. Blank. Fermentation and purification strategies for the production of betulinic acid and its lupane‐ type precursors in *Saccharomyces cerevisiae*. Biotech. Bioeng. 114, 2528–2538 (2017) https://doi.org/10.1002/bit.26377

In addition, this first‐generation *P. tricornutum* strain can be grown photoautotrophically and is rich in fucoxanthin and long‐chain polyunsaturated fatty acids (lcPUFAs), adding value to the platform. Further optimizations in genetics and culture conditions, and extraction of multiple value‐added molecules are expected to substantially improve the overall process economics. In Figure 5, we report the extraction procedure of triterpenes and a proposed biorefinery approach. Sources for this analysis can be found in Table S4. The extraction procedure for triterpene is the same for yeast, and microalgae and estimated costs are equal. Refining the microalgal biomass from this purification procedure allows the separation of other valuable molecules. Examples of these coproducts include eicosapentaenoic acid (market estimated to reach $3.79 billion by 2022), carotenoids such as fucoxanthin (currently $95 million global market) and phytosterols such as brassicasterol ($62.5 million global market by 2020). The defatted biomass (protein and sugars) can still be used as a low‐value animal food. Therefore, further optimizations in genetics and culture conditions, and extraction of multiple value‐added molecules, are expected to increase the competitiveness of microalgal platforms.

### Triterpenoid production affects the expression levels of native sterol metabolism enzyme transcripts in the LjLUS‐25 transformant line

In eukaryotic photosynthetic organisms, different terpenoid classes are produced in different cellular compartments. Both carotenoids and sterols are produced from the same precursor isopentenyl pyrophosphate (IPP), but the former is made in the chloroplast while the latter is made in the ER. Higher plants and *P. tricornutum* contain both the cytosolic MVA and the chloroplast‐localized MEP pathway (Figure 1). While both pathways produce IPP, there appears to be no or minimal IPP interchange between the cytosol and chloroplast in higher plants (Rodríguez‐Concepción, 2006). Indeed, disruption of key genes in either the MVA or MEP pathway results in distinct and severely deleterious phenotypes in *A. thaliana* (Hsieh and Goodman, 2005; Suzuki *et al*., 2004). Green algae such as *C. reinhardtii* only contain the chloroplast‐localized MEP pathway which supplies IPP for both the ER‐localized sterol biosynthesis and photosynthetic pigments.

The introduction of a non‐native OSC such as the LjLUS in *P. tricornutum* could potentially affect sterol biosynthesis or the IPP‐producing pathways. Therefore, we measured the expression of a selected set of sterol biosynthetic enzymes, including enzymes from the chloroplast‐localized MEP pathway and cytosolic MVA pathway, by qPCR to determine whether ectopic *LjLUS* influences native sterol biosynthesis expression.

Samples for RNA extraction were taken from cultures of wild type and LjLUS‐25 line during 5 days of culturing. The algae started entering stationary phase by the fifth day (Figure S4). It was immediately apparent that expression of sterol biosynthesis enzymes is higher during exponential phase and nearly all investigated genes reached their expression minimum on day 5. This holds true for both the wild type and the LjLUS overexpression line and correlates well with the lupeol and brassicasterol levels measured (Figure 4a–b).

**Figure 6:**
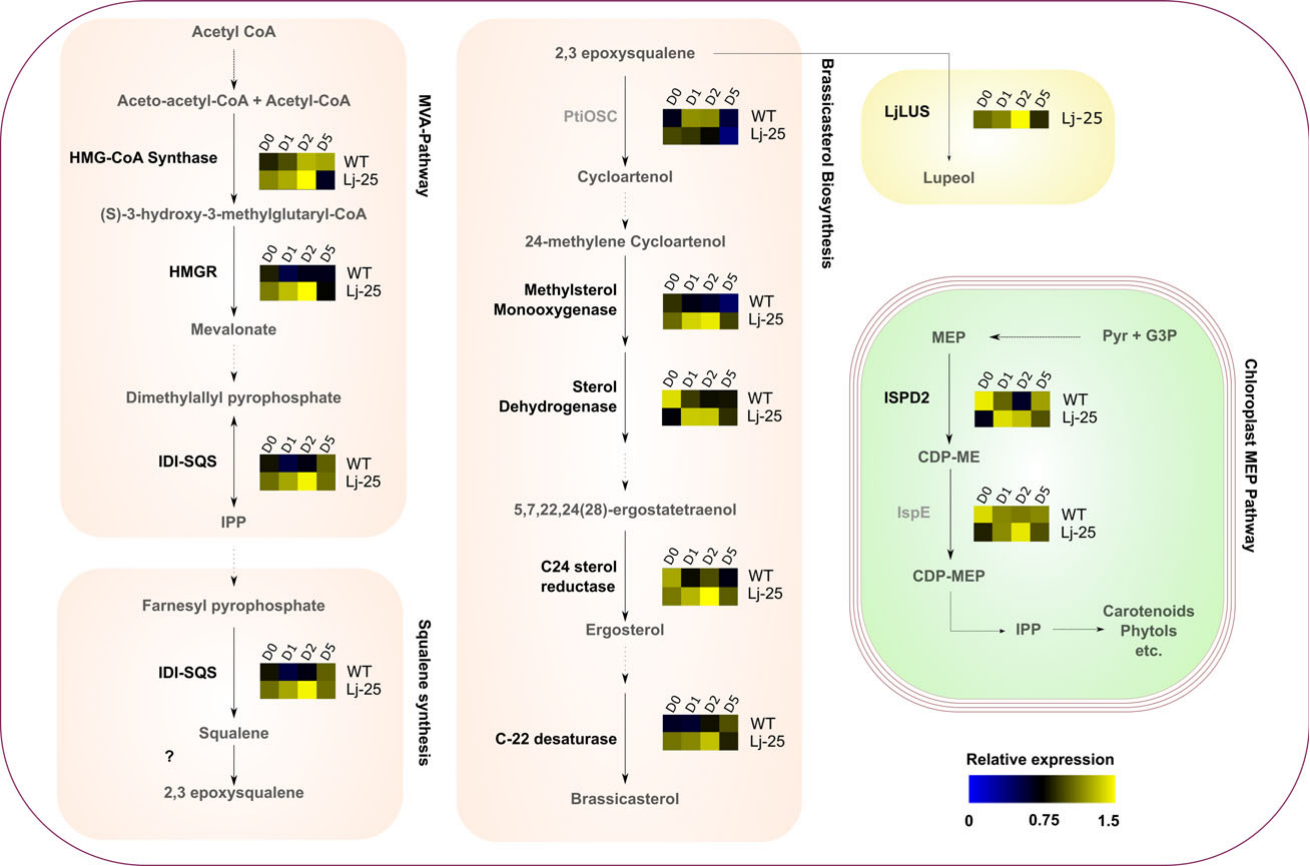
mRNA expression of sterol biosynthesis enzymes during a five‐day time course in the wild type (WT) and the LjLUS‐25 line. Schematic representation of sterol metabolism in *Phaeodactylum tricornutum*, indicating the gene selected for mRNA expression analysis and relative heat map for WT and LjLUS‐25 line. Values are the expression relative to the geometric average of the *RP3a* and *UBQ* reference genes. Transcripts not significantly different between wt and LjLUS are indicated in grey. Enzymes involved in the cytosolic MVA pathway: HMGS: hydroxymethylglutaryl‐CoA synthase; HMGR: 3‐hydroxy‐3‐methyl‐glutaryl‐coenzyme A reductase; IDI‐SQS: isopentenyl diphosphate isomerase‐squalene synthase. Enzymes involved in brassicasterol biosynthesis: PtOSC: oxidosqualene cyclase, methylsterol monooxygenase, sterol dehydrogenase, C24 sterol reductase, C22 sterol desaturase. Enzymes involved in the plastidial MEP pathway: ISPD2: 2‐C‐methyl‐d‐erythritol 4‐phosphate cytidylyltransferase, ISP‐E: 4‐diphosphocytidyl‐2‐C‐methyl‐d‐erythritol kinase. In the yellow box, the non‐native lupeol synthase from *Lotus japonicus*, introduced in our engineered strain LjLUS25. Dashed lines indicate multiple reactions. Gene accession numbers are mentioned in the results section. Precursor abbreviations: IPP: Isopentyl pyrophosphate, MEP: 2‐*C*‐methyl‐d‐erythritol 4‐phosphate, CDP‐ME: 4‐diphosphocytidyl‐2‐*c*‐methylerythritol, CDP‐MEP: 4‐diphosphocytidyl‐2‐*c*‐methyl‐d‐erythritol 2‐phosphate, DMAP: dimethylallyl pyrophosphate, G3P: glycerol 3‐phosphate, Pyr: pyruvate.

Expression of some of the mevalonate pathway encoding genes increased markedly in the LjLUS‐25 line compared to the wild type as shown in Figure 6. The statistical significance of the investigated genes was tested using a repeated measures ANOVA model (Table S5). Genes coding for key enzymes such as HMGR (*Phatr3_J16649*) and the IPP isomerase–squalene synthase fusion enzyme (*Phatr3_EG02290*) are consistently up‐regulated, while HMG‐CoA synthase (*Phatr3_J16649*) increased only slightly.

Since the levels of brassicasterol are lower in the LjLUS‐25 line, we expected to see marked up‐regulation of genes involved in brassicasterol synthesis in an attempt to compensate. Surprisingly, no up‐regulation was seen for the PtOSC transcript (*Phatr3_EG02293*+ *Phatr3_J46726* fusion) even though it competes with the LjLUS enzyme for 2,3‐oxidosqualene. However, enzyme transcripts downstream of PtOSC show up‐regulation. This includes a methylsterol oxygenase (*Phatr3_J10852*), a sterol dehydrogenase (*Phatr3_J48864*), a C24 sterol reductase (*Phatr3_J48260*) and C22 reductase (*Phatr3_J51757*) with all of these enzymes showing a similar gene expression pattern. The MEP pathway transcripts *IspE* (*Phatr3_EG02383*) and *ISPD2* (*Phatr3_J21829*) did not show a consistent up‐regulation pattern in transgenic lines and its role in sterol biosynthesis, if any in *P. tricornutum*, remains unclear. Overall these results suggest that, as in higher plants, the MVA pathway is the major source of IPP for sterol biosynthesis and triterpenoid production in *P. tricornutum*. This is in agreement with earlier studies, which showed that terpenoids in the *P. tricornutum* cytosol and chloroplast do not have the same source of IPP (Cvejić and Rohmer, 2000).

### Introducing a CYP716A12 cytochrome P450 and a P450‐NADPH reductase leads to the oxidation of lupeol to BA

In several higher plant species (e.g. *Betula platyphylla*), lupeol is oxidized at the C28 position by a cytochrome P450‐dependent monooxygenase (CYP) to form BA (Figure 1) (Hamberger and Bak, 2013). Recently, a number of studies have reported enzymes of the CYP716A family to be responsible for this modification (Andre *et al*., 2016; Carelli *et al*., 2011; Fukushima *et al*., 2011; Moses *et al*., 2014). Based on the literature, we selected the *Medicago truncatula* CYP716A12 and CPR for expression in *P. tricornutum*. In‐house activity assays in yeast confirmed that the *M. truncatula* CYP716A12 enzyme is a multifunctional enzyme with β‐amyrin 28‐oxidase, α‐amyrin 28‐oxidase and lupeol 28‐oxidase activities.

Cytochrome P450 enzymes work in tandem with specific cytochrome P450 reductases (CPR), which shuttle electrons from NAD(P)H to the cytochrome P450. The CPR–P450 interacting domains are well conserved, as demonstrated by the ability of CPRs from different species to at least partially complement in functional terms. Although eukaryotes such as *S. cerevisiae* and *P. tricornutum* contain a native CPR, CYP enzymes usually require a CPR from the same or a related species for maximum activity when introduced into a heterologous host, to ensure an adequate supply of electrons to the CYP and potentially minimizing the release of reactive oxygen species (Jennewein *et al*., 2005; Jensen and Møller, 2010; Kim *et al*., 2005, 2009; Zangar *et al*., 2004). This reduction step can be rate limiting, and increased catalytic efficiency has been obtained by fusing one or more CPRs directly to the CYP enzymes (Leonard *et al*., 2006; Munro *et al*., 2007; Schückel *et al*., 2012).

In this work, we tested the expression in *P. tricornutum* of the *M. truncatula* CYP716A12 (MtCYP716A12), coexpressed along with the *M. truncatula* CPR (MtCPR). In order to increase catalytic efficiency of the CYP–CPR system, the MtCYP716A12 sequence was fused to its native MtCPR coenzyme. We therefore employed two approaches:

1. A cotransformation approach wherein *P. tricornutum* was simultaneously transformed with the LjLUS + zeocin resistance gene marker (*ble* ^r^), MtCYP716A12, and MtCPR coding sequences.
2. An iterative approach, where *P. tricornutum* was first transformed with an OSC construct and the *ble* ^*r*^ gene marker. The resulting lines were screened for lupeol production, and the best strain was further transformed with a fused version of MtCYP716A12‐CPR and the nourseothricin resistance selection marker (*nat* ^*r*^).

For the cotransformation approach, the native genes encoding LjLUS, MtCYP716A12 and MtCPR were integrated into the genome of *P. tricornutum* in a single transformation, using three plasmids (Figure S1).

In the iterative approach, we fused the *M. truncatula* CYP716A12 and CPR enzyme, following a strategy inspired by the work of Leonard and Koffas, where they functionally expressed the *Glycine max* P450 isoflavone synthase fused to a CPR from *C. roseus* in *E. coli* (Leonard and Koffas, 2007; Leonard *et al*., 2006). The full coding sequence of MtCYP716A12 without the stop codon was fused in‐frame to the MtCPR coding sequence with the exclusion of the first 71 amino acids, which are predicted to correspond to the transmembrane domain (Δ71CPR). The membrane anchor was deleted to avoid unwanted membrane insertion or association, which could compromise the enzyme activity. The two coding sequences were spaced by an additional short stretch of DNA coding for a flexible linker λ (GSTSSGSG), to prevent the formation of secondary structures, which could disrupt the native 3D structure of the two enzymes. The resulting final coding sequence, MtCYP716A12‐λ‐Δ71CPR, was cloned under the control of the *P. tricornutum FCPA/LHCF1* promoter. This vector was used to transform the best lupeol‐producing line: LjLUS‐25 (Figure 2).

For both approaches, the transformant lines were grown on selective solid medium, then transferred into liquid medium, and metabolites analysed by GC‐MS in early stationary phase.

**Figure 7:**
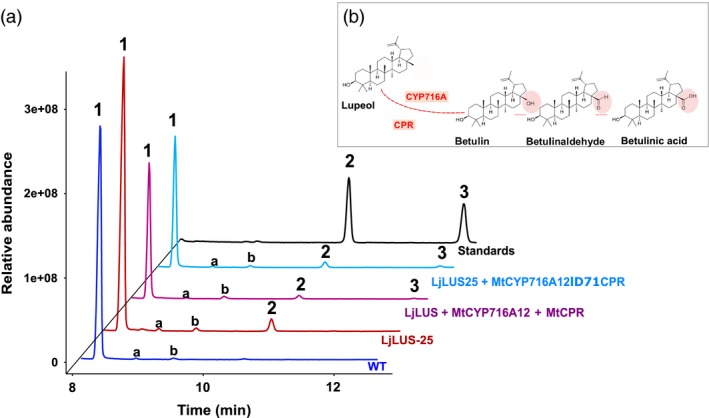
Betulin production in *Phaeodactylum tricornutum* transformant lines. (a) GC‐MS analysis of WT (blue), LjLUS‐25 containing only the *Lotus japonicus* lupeol synthase (LjLUS) (red); transformant line with constructs harbouring *L. japonicus* lupeol synthase (LjLUS), *Medicago truncatula* cytochrome P450 (MtCYP716A12) and cytochrome P450 reductase (MtCPR) in WT background (purple); transformant line with construct harbouring fused P450‐reductase protein MtCYP716A12λΔ72CPR in LjLUS‐25 background (light blue); standards (black). Peak as indicated 1. Brassicasterol; 2. Lupeol; 3. Betulin. (b) Detail of CYP716A enzyme reaction. The oxidation at C28 leads to betulin intermediate, which is converted to betulin aldehyde, and betulinic acid.

All combinations and strategies resulted in transformant lines that produced betulin, the first intermediate in BA production (Figure 7). Peak identity was confirmed by comparing the electron impact fragmentation patterns with those of known standards (Figure S3E–I). Of the thirty independent lines that were screened per transformation, one cotransformation line and two fusion lines showed clear peaks for betulin. This intermediate is also the main product identified in recombinant yeast expressing this enzyme (Fukushima *et al*., 2011). As shown in Figure 7, the peak of betulin is very low compared to lupeol and brassicasterol, making the quantification by GC‐analysis challenging.

In the cotransformation approach, we showed that *P. tricornutum* is able to integrate three different plasmids in a single cotransformation event and functionally express the transgenes. This approach was mainly aimed to reduce time and cost of transformation, compared to the stepwise transformation, and to side‐step the limited resistance markers available for selection, with only two well‐characterized antibiotic selection markers currently available for *P. tricornutum* (*ble* ^*r*^ and *nat* ^*r*^). This compares favourably to *C. reinhardtii* where the frequencies for the successful expression of a single transgene, such as GFP or luciferase, from the nuclear genome vary around 10%, and it has been shown that strong expression can only be obtained by translation linkage with an antibiotic resistance marker or the usage of mutagenized strains (Fuhrmann *et al*., 2004; Neupert *et al*., 2009; Rasala *et al*., 2013). This would preclude a similar approach being applied in *C. reinhardtii* without extensive screening as performed in Lauersen *et al*. (2016).

In the iterative approach, we demonstrated the possibility of successfully applying protein engineering approach to redesign the *M. truncatula* CYP716A12‐CPR system, for lupeol modification. This allowed us to economize on promoters and antibiotic markers in systems with limited molecular biology tools. Although more effort is needed to improve the efficiency of betulin and BA production, these results establish the feasibility of using existing genetic engineering strategies for plant pathway reconstitution in *P. tricornutum*, making this microorganism a promising system for industrial biotechnology.

### Growth performance analysis and scaling up process

The repurposing of sterol pathway intermediates and random nuclear genome insertion could have a detrimental effect on growth speed and biomass accumulation of the transformant lines. Therefore, we compared growth performances of lupeol‐ and betulin‐producing lines with the wild‐type strain in an Algem^®^ labscale photobioreactor. As shown in Figures S4 and S5, no significant differences were observed between the wild type and any of the transformant strains. This suggests that the introduction of the OSC, cytochrome P450 and reductase genes did not affect cell growth and that the products of these enzymes are well tolerated at the levels produced.

Following these smaller‐scale trials in Algem^®^ photobioreactors, we compared the biomass productivity and lupeol content of three transgenic strains: AtLUS‐6, LjLUS‐25 and LjLUS + MtCYP716A12 + MtCPR in a fence‐style tubular photobioreactor (PBR). Transgenic cultures were grown in a 550‐L working volume photobioreactor, which offers control over pH, lighting, improved agitation and CO_2_ delivery. Cultures were monitored over a period of 25 days for the first harvest, once they reached early stationary phase, with a cell density in line with the flask shake cultures.

In this large‐scale trial, despite displaying expected growth kinetics, line LjLUS‐25 line did not recover following the initial harvest (Figure S6). This was unexpected as growth curves did not show any difference in behaviour compared to the two other lines tested at the large scale. This was likely due to an unidentified technical performance issue in the bioreactor system immediately prior, during or postharvesting, since one of the well‐performing lines (LjLUS + MtCYP716A12 + MtCPR) is a derivative of the LjLUS25. The other two lines showed the expected growth performance, allowing multiple harvests (30 days for AtLUS6 and 20 days for LjLUS + MtCYP716A12 + MtCPR). We performed a small‐scale extraction from the dry biomasses harvested from this trial, using an amount of dry biomass equivalent to approximately 1 L culture. Table S2 shows the lupeol quantification for the AtLUS6 and LjLUS‐25 lines, as expected from the growth fitness, we extracted lower amount of lupeol in LjLUS‐25 line, compared to AtLUS6; this result might be due to the status of LjLUS‐25 culture at the time of harvest. Further analysis on the scale‐up system and the extraction optimization from dry biomass will enable us to investigate the feasibility of this algal system for commercialization.

## Conclusions

Herein, we have demonstrated the feasibility of introducing plant transgenes in *P. tricornutum,* for the production of high‐value sapogenins, without evident impact on growth and fitness. We show for the first time the possibility of introducing in *P. tricornutum* a plant pathway that requires the expression of genes corresponding to three different membrane‐bound enzymes: a lupeol synthase, a P450 enzyme and a NADPH reductase. This is the most complex terpenoid pathway expressed to date in photosynthetic microorganisms. Previously, mono‐ and sesquiterpenoid production has been achieved in these organisms through the introduction of single enzymes. We have, therefore, demonstrated the feasibility and relative ease in terms of screening and genetic approach, of introducing multiple heterologous enzymes, to reconstitute a plant secondary metabolite pathway in a microalga. The results from this study provide novel insights on the regulation of the sterol biosynthesis pathway, which can be employed in future terpenoid pathway engineering. Gene expression patterns suggest that there is limited precursor availability, which could be overcome by the overexpression of key MVA pathway enzymes or the introduction of heterologous enzymes that bypass native regulation. A prime candidate would be the HMGR enzyme, as it has been shown to result in higher sterol and triterpenoid levels in several organisms (Chappell *et al*., 1995; Moses *et al*., 2014).

Further investigation to test the feasibility of having multiple enzyme fusions or using a synthetic scaffold approach to create metabolon‐like complexes (Dueber *et al*., 2009) will allow improved triterpenoid production in *P. tricornutum*. The BA pathway is well suited for the latter approach, as the plant CYP's localize to the membrane and can assemble in dynamic complexes (Bassard *et al*., 2017). Moreover, we showed the feasibility of growing this microalga at large scale. Although there is still substantial room for improving the product yield through bioprocess optimization and metabolic pathway engineering in *P. tricornutum,* this first‐generation strain and our results open a new perspective and are instructive with regard to the establishment of a new realm of investigations towards industrial biotechnology exploitation for this photosynthetic microorganism. Substantial advances will need to be made for algae with regard to both gene expression control and cultivation before this platform could become commercially feasible for the production of small molecules. To date, the regulation of the lipid and sterol metabolism of diatoms is largely unknown; a recent study has shown that sterol biosynthesis can be chemically induced in a lipid‐independent manner (Prioretti *et al*., 2017). This study, therefore, represents pioneering work validating the potential of these eukaryotic microalgae as a chassis for the photo production of value‐added molecules. Future studies, that include enzyme screening and engineering, modification on the carbon sink, semi‐continuous batch system analyses, as well as use of a biorefinery approach for extraction of value‐added molecules such as fucoxanthin and long‐chain PUFAs, could likely improve the overall productivity and economics towards the establishment of a renewable triterpenoid‐producing platform.

## Materials and methods

### Strain and growth conditions

*Phaeodactylum tricornutum* CCAP1055/1 was grown in F/2 medium. Cells were transferred from plate to 5 mL of F/2 medium for 5 days and then inoculated in 25 mL as preculture until reaching mid‐log phase (~3 days) and then transferred to fresh medium with a 1:5 dilution. Cells were all grown at 20 °C in New Brunswick™ Edison, NJ, USA incubator agitated at 120 rpm under white fluorescent light (60 μmol/m^2^/s).

### Algem growth analysis

For strain performance analysis, cells were grown in Algem^®^ labscale photobioreactor systems (Algenuity, Stewartby, UK). Light was set to 150 μmol photons/m^2^/s using the ‘sunlight’ LED profile with temperature and shaking kept constant at 21 °C and 120 rpm, respectively. The pH was kept at 8 by controlled bubbling with 3% CO_2_ in air. OD_740 nm_ measurements were taken every 10 min using the in‐built sensor.

To monitor lupeol and brassicasterol productivity and for gene expression analysis, cells were subcultured twice until they reached mid‐exponential phase and then inoculated at a cell culture density of 2 × 10^6^ cells/mL in fresh in F/2 medium and grown in Algem^®^ labscale photobioreactors. Light was set to 400 μmol photons/m^2^/s using the ‘sunlight’ LED profile with temperature and shaking pH control and OD measurements as described above.

### Large‐scale cultivation

For large‐scale growth, a fence‐style photobioreactor (Bouygues Energies and Services, Manchester, UK) with a working volume of approximately 550 L was used. A photostage consisting of an array of horizontal polycarbonate 50‐mm‐diameter tubes (36 tubes in total, in a six tube manifold formation) was illuminated at a light intensity of 450 μmol photons/m^2^/s, by eight 600‐W high‐pressure sodium lamps. Continuous circulation of the culture via centrifugal pump (up to 30 m^3^/h) was allowed by stainless steel (316S) pipework connected to a holding tank (200 L). The holding tank was continually sparged with a flux of air (~10 L/min) for oxygen removal, while a pH‐stat system controlled carbon dioxide delivery. Culture monitoring, which included oxygen saturation, pH, conductivity and temperature measurement was performed via a Rheinland‐Pfalz, DE Profilux 3 interface. For media composition, F/2 nutrients (Varicon Aqua, Worcester, UK) were used in combination with artificial sea water (Instant Ocean, Blacksburg VA, USA).

### Plasmid design and transformation

pPhaT‐1 (GenBank accession number: AF219942) vector including *AtLUS* (GenBank accession number: NP178018.1) and ble^r^ marker gene sequence was a gift from the group of Alain Goossens (PSB‐VIB, Ghent, Belgium). Sequences for *LjLUS*:* OSC3* (GenBank accession number: AB181245), *MtCYP716A12* (GenBank accession number DQ335781), *MtCPR*: MTR_3g100160 (NCBI reference sequence XM_003602850.2), *nat* ^r^: (GenBank accession number: X73149) were synthesized as gBLOCKs (IDT, Leuven, Belgium) and introduced by Golden Gate cloning in final recipient plasmids, according to the design rules of the Open Plant standard (Patron *et al*., 2015) (Figure S1). Where necessary, PCRs were performed with Q5 polymerase (New England Biolabs, Hitchin, UK). All primers used are listed in Table S3.

For the fusion of MtCYP716A12 with MtCPR, firstly the sequence encoding for the predicted membrane‐spanning domain of MtCPR (1–71 AA, 1–213 bp) was removed from the original sequence and a sequence coding for a flexible linker GSTSSGSG (λ) was added in‐frame to the remaining sequence (72–692AA, 214–20 179 bp) creating a cDNA encoding for a λΔ71CPR. The cDNA coding for MtCYP716A12 without the stop codon was added in‐frame by Gibson assembly to the sequence λΔ71CPR, in order to obtain a chimeric cDNA encoding a fusion version of MtCYP716A12λΔ71CPR.

Biolistic transformation of *P. tricornutum* was performed according to methods previously described (Kroth, 2007b). Bombarded cells were transferred onto F/2 medium (with 1.7% of NaCl) agar plates containing either 75 μg/mL zeocin (Invivogen, San Diego, CA, USA) or 50 μg/mL nourseothricin (Carl Roth, Karlsruhe, DE, Roth, Germany) depending on selection method. The selective medium plates were placed in 24‐h illumination under fluorescent lights (60 μmol/m^2^/s) and incubated at 20 °C for 3 weeks. Individual antibiotic‐resistant colonies were transferred to fresh selective plates and subsequently transferred into liquid medium.

### GC‐MS analyses

All cell lines were grown until early stationary phase (~5 days) and harvested by centrifugation 4200× *g* for 10 mins at 4 °C. For screening of transformant lines, beta methyl cyclodextrin (Sigma‐Aldrich, Schnelldorf, DE) was added to 25‐mL cell culture to a final concentration of 5 mm, 24 h prior to the harvest, cells were collected by centrifugation, and supernatant containing beta methyl cyclodextrin was extracted with pentane and analysed as previously described (Moses *et al*., 2015).

For quantification, analysis of lupeol cell pellets derived from 100 mL of cells was lysed by saponification and analysed as previously described (Zhou *et al*., 2016). Derivatization was performed using *N*‐methyl‐*N*‐(trimethylsilyl)trifluoroacetamide (Sigma‐Aldrich, Gillingham, UK). Standards were obtained from ExtrasyntheseSAS (Genay Cedex, France) and Molekula, Newcastle (UK).

For betulin detection, after quick screen method with beta methyl cyclodextrin, as described above, 400 mL of the positive lines were grown until stationary phase (~5 days) and harvested and processed as described above for lupeol quantification analysis.

All GC‐MS samples were run on a Thermo TRACE‐GC DSQII quadrupole with a SLB‐5MS column (Sigma‐Aldrich, UK). The injector temperature was set to 280 °C and the MS‐transfer line was set to 320 °C. Oven temperature was set to 120 °C and subsequently increased to 290 °C (50 °C/min ramp), after which it was ramped to 300 °C at (1 °C/min).

### Copy number analysis and mRNA expression analysis

RNA and DNA were extracted using TriReagent (Thermo, Waltham MA, USA) following the manufacturer's protocol. First‐strand cDNA synthesis was performed using MMuLV (New England Biolabs) with 500 ng of total RNA as input. For gene expression analysis, cDNA was diluted 1/20 and the RP3a and UBQ genes were used as reference genes as described earlier (Matthijs *et al*., 2017). The same primers were used as normalizers for the gDNA CNV analysis. Quantitative PCR was performed with JumpStart SYBR green ReadyMix (Sigma‐Aldrich, UK). Analysis was performed using the ΔΔCt method as implemented in CFX Manager (Livak and Schmittgen, 2001) (Bio‐Rad, Watford, UK). All primers used are listed in Table S3. ANOVA and Tukey tests were computed in R 3.43 using the agricolae 1.2‐8 package. The significance of correlation was determined by calculating a *t*‐statistic using the formula: $t=\left|r\right|\sqrt{\frac{n-2}{1-r^{2}}}$.

## Conflicts of interest

At the time of the study, SD, JS, GS, GPL, APS and MM were employed by Algenuity, a division of Spicer Consulting Limited, UK, which manufactures the Algem photobioreactor used for parts of this study.

## Supporting information


**Figure S1** Illustration of plasmids used for *P. tricornutum* transformation.
**Figure S2** Molecular Phylogenetic tree of selected plant OSCs with the highest log likelihood by Maximum Likelihood method of Whelan and Goldman model [1].
**Figure S3** GC‐MS ionization pattern for lupeol and betulin peaks.
**Figure S4** Growth performance of the LUS transgenic lines in Algem^®^ photobioreactors.
**Figure S5** Growth performance of the Betulin transgenic lines in Algem^®^ photobioreactors.
**Figure S6** Growth performance in 550L tubular PBR.
**Table S1** Quantification of lupeol accumulation in the best performing transformant lines.
**Table S2** Scale‐up conditions and specification.
**Table S3** Oligonucleotides used in this study.
**Table S4** Sources for the economic analysis.
**Table S5** Repeated measures significance of the genes shown in Figure 6.
