# Supplementary material for: Engineering the unicellular alga Phaeodactylum tricornutum for high‐value plant triterpenoid production
Source: Plant Biotechnol J. 2018 Jun 19;17(1):75–87. doi: 10.1111/pbi.12948 (PMC6330534; doi:10.1111/pbi.12948)
Supplement: Supplementary file 1 — Figure S1 Illustration of plasmids used for P. tricornutum transformation. Figure S2 Molecular Phylogenetic tree of selected plant OSCs with the highest log likelihood by Maximum Likelihood method of Whelan and Goldman model [1]. Figure S3 GC‐MS ionization pattern for lupeol and betulin peaks. Figure S4 Growth performance of the LUS transgenic lines in Algem® photobioreactors. Figure S5 Growth performance of the Betulin transgenic lines in Algem® photobioreactors. Figure S6 Growth performance in 550L tubular PBR. Table S1 Quantification of lupeol accumulation in the best performing transformant lines. Table S2 Scale‐up conditions and specification. Table S4 Sources for the economic analysis. [file PBI-17-75-s002.pdf]

Supplementary Tables

Strain	ng lupeol · 10^7cell -1	mg lupeol· L-1	mg lupeol· g dry weight-1	Group
LjLUS-18	13.97 ± 5.9	0.024 ± 0.003	0.05 ± 0.0032	b
LjLUS-20	35.06 ± 6.5	0.046 ± 0.015	0.11 ± 0.010	a
LjLUS-25	30.01 ± 4.0	0.044 ± 0.009	0.10 ± 0.021	a
AtLUS-6	8.7 ± 1.9	0.005 ± 0.0007	0.013 ± 0.0014	b

Supplementary table 1. Quantification of lupeol accumulation in the best performing transformant lines. Lupeol extracted from cell pellet of best performing strains, selected from previous semi-quantitative analyses. Quantities were normalized per cell number, per liter of culture, and per gram of dry weight. Errors represent standard deviation from three biological replicates. An ANOVA test revealed significant differences between the tested lines $F(3, 8) = 44$, $p < .0001$. The letters in group represent the statistically different groups of a Tukey Post hoc test for mg lupeol per gram of dry weight at $\alpha = 0.01$, the minimum significant difference was 0.042

Strain	Total harvest (L)	Cell /mL at the harvest	Total cell dry weight (g)	µg of lupeol/g dry weight	Group
AtLUS-6	430	1.32*10^7	98	13 ± 0.15	a
LjLUS-25	258	1.25*10^7	58	5.8 ± 0.45	b
LjLUS+MtCYP716A12+MtCPR	466	1.54*10^7	88.64	4.68± 0.22	c

Supplementary table 2. Scale-up conditions and specification. Growth parameters of the 550L tubular photobioreactor (PBR) used for pilot scale production. Strain, liter of biomass harvested, cell densities at harvest and total cell dry weight harvested are shown. Lupeol quantification per gram dry weight was also determined. Errors represent standard deviation from three biological replicates. An ANOVA test revealed significant differences between the tested lines $F(2, 6) = 671$, $p < .0001$. The letters in group represent the statistically different groups of a Tukey Post hoc test for mg lupeol per gram of dry weight at $\alpha = 0.01$, the minimum significant difference was 1.10

Product	Market Valuation (Millions)	Reached by year:	Source
EPA	3 790	2022	globenewswire.com/news-release/2016/03/30/823963/0/en/EPA-DHA-Omega-3-Ingredients-Market-Size-worth-USD-3-79-Billion-by-2022-Global-Market-Insights-Inc.html
Fucoxhantin	95	2016	ktvn.com/story/36579624/fucoxanthin-market-growth-analysis-opportunities-forecasts-report-till-2022
Phytosterols	62.5	2020	marketsandmarkets.com/Market-Reports/phytosterols-market-223259471.html

Supplementary table 4: Sources for the economic analysis

Supplementary Figures

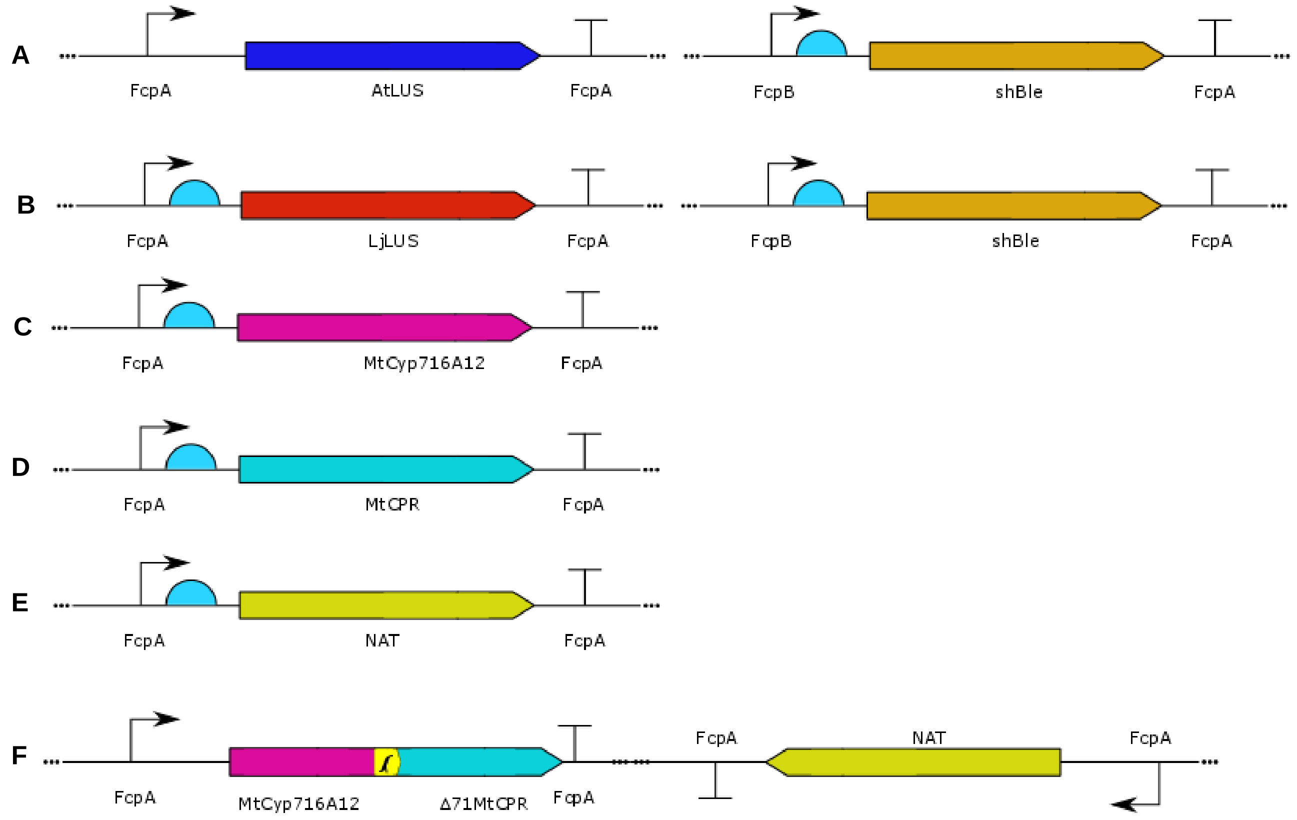

Supplemental Figure 1. Illustration of plasmids used for *P. tricornutum* transformation. All expressed gene were under control of native fucoxanthin chlorophyll a/c binding protein A (*FCPA/LHCF1*) promoter. **A.** Expression vector containing lupeol synthase from *A. thaliana* (*AtLUS*) and zeocin resistance (*ble*^{r}) encoding genes **B.** Expression vector containing lupeol synthase from *L. japonicus* (*LjLUS*) and zeocin resistance (*ble*^{r}) encoding genes. **C.** Expression vector containing *M. truncatula* cytochrome P450 (*MtCYP716A12*) encoding gene. **D.** Expression vector containing *M. truncatula* cytochrome P450 reductase (*MtCPR*) encoding gene. **E.** Expression vector containing nourseothricin (*nat*^{r}) resistance encoding gene. **F.** Expression vector containing engineered encoding gene of *M. truncatula* cytochrome P450 fused to *M. truncatula* cytochrome P450 reductase (*MtCYP716A12λΔ71MtCPR*) and nourseothricin (*nat*^{r}) resistance encoding gene.

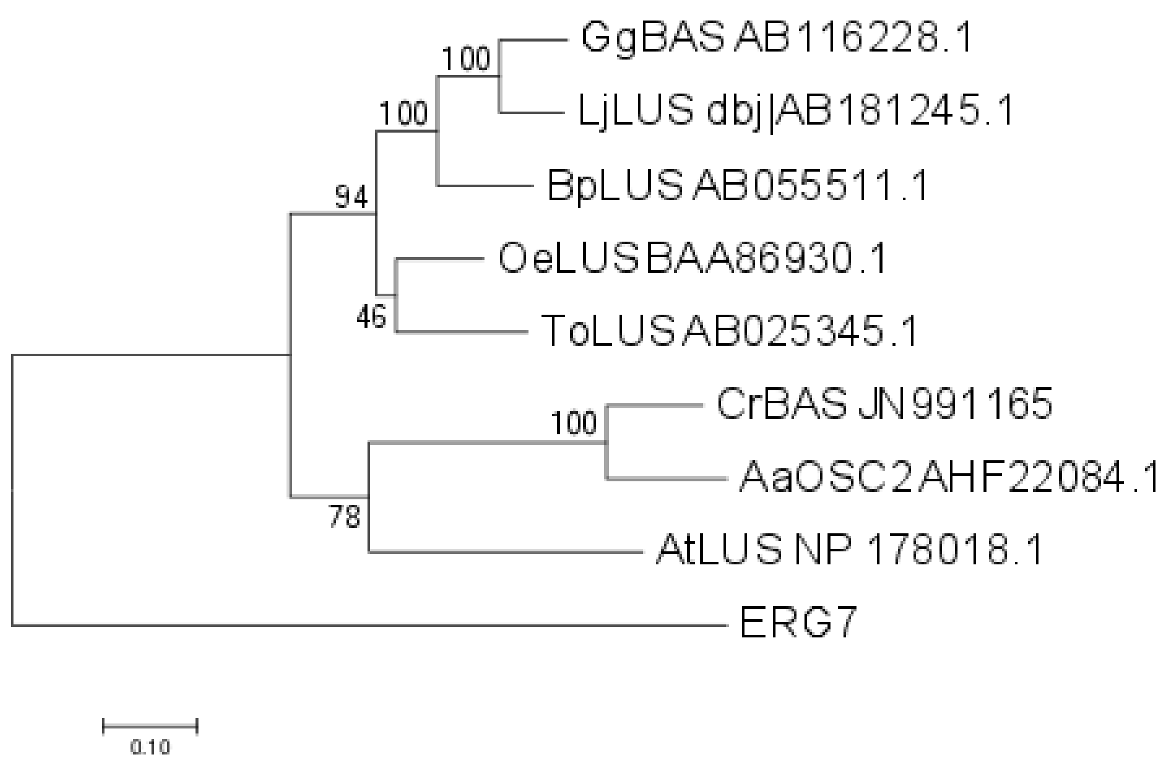

Supplemental Figure2: Molecular Phylogenetic tree of selected plant OSCs with the highest log likelihood by Maximum Likelihood method of Whelan and Goldman model [1].

The percentage of trees in which the associated taxa clustered together is shown next to the branches. Initial tree(s) for the heuristic search were obtained automatically by applying Neighbour-Join and BioNJ algorithms to a matrix of pairwise distances estimated using a JTT model, and then selecting the topology with superior log likelihood value. The tree is drawn to scale, with branch lengths measured in the number of substitutions per site. All positions with less than 95% site coverage were eliminated. That is, fewer than 5% alignment gaps, missing data, and ambiguous bases were allowed at any position. There were a total of 708 positions in the final dataset. Evolutionary analyses were conducted in MEGA7 [2]. AaOSC2: *Artemisia annua*, AtLUS: *Arabidopsis Thaliana*, BpLUS: *Bupleurum lanceolate*, CrBAS: *Catharantus roseus*, ERG7: *S. cerevisiae* Lanosterol cyclase, GgBAS: *Glycyrrhiza Glabra*, LjLUS: *Lotus japonicus* OeLUS: *Olea europea*, ToLUS: *Taraxacum officinale*

1. Whelan, S. and Goldman, N. (2001). A general empirical model of protein evolution derived from multiple protein families using a maximum-likelihood approach. Molecular Biology and Evolution 18:691-699.

2. Kumar S., Stecher G., and Tamura K. (2016). MEGA7: Molecular Evolutionary Genetics Analysis version 7.0 for bigger datasets.Molecular Biology and Evolution 33:1870-1874.

3. Felsenstein J. (1985). Confidence limits on phylogenies: An approach using the bootstrap. Evolution 39:783-791

Supplemental Figure 3: GC-MS Electron impact ionization pattern for lupeol and betulin peaks. **A.** Lupeol peak from LjLUS-25. **B.** Lupeol peak of standard. **C.** Lupeol peak of AtLUS-6. **D.** 2-3β hydroxylupane from AtLUS-6. **E.** Lupeol peak of LjLUS+MtCYP716A12+MtCPR. **F.** Betulin peak from LjLUS+MtCYP716A12+MtCPR. **G.** Lupeol peak of LjLUS_MtCYP716A12λΔ71MtCPR. **H.** Betulin peak of LjLUS_MtCYP716A12λΔ71MtCPR. **I.** Betulin peak of standard.

SF3-A:Ionization pattern of lupeol peak from LjLUS-25 transformant line

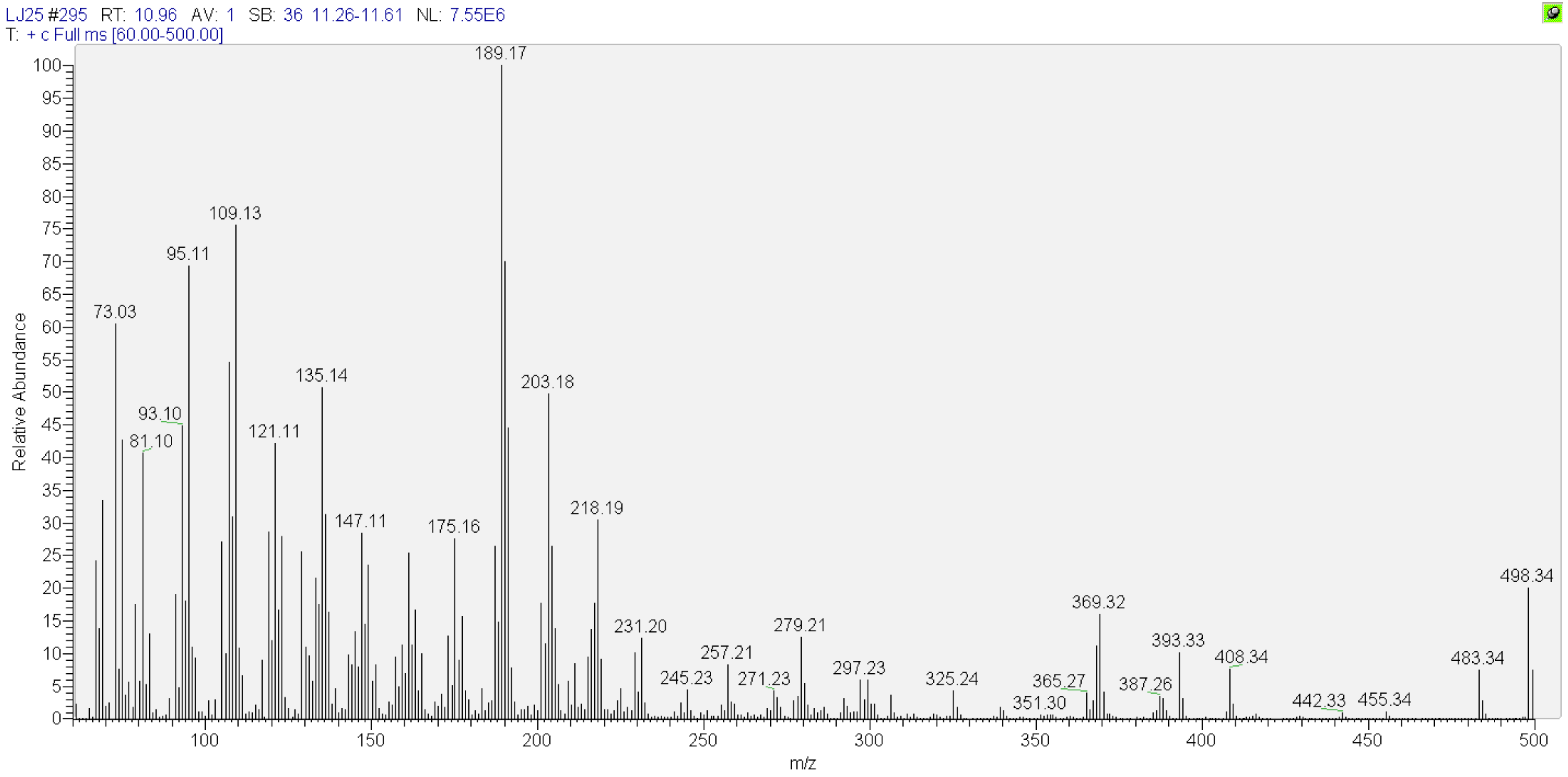

SF3-B: Ionization pattern of lupeol peak from standard

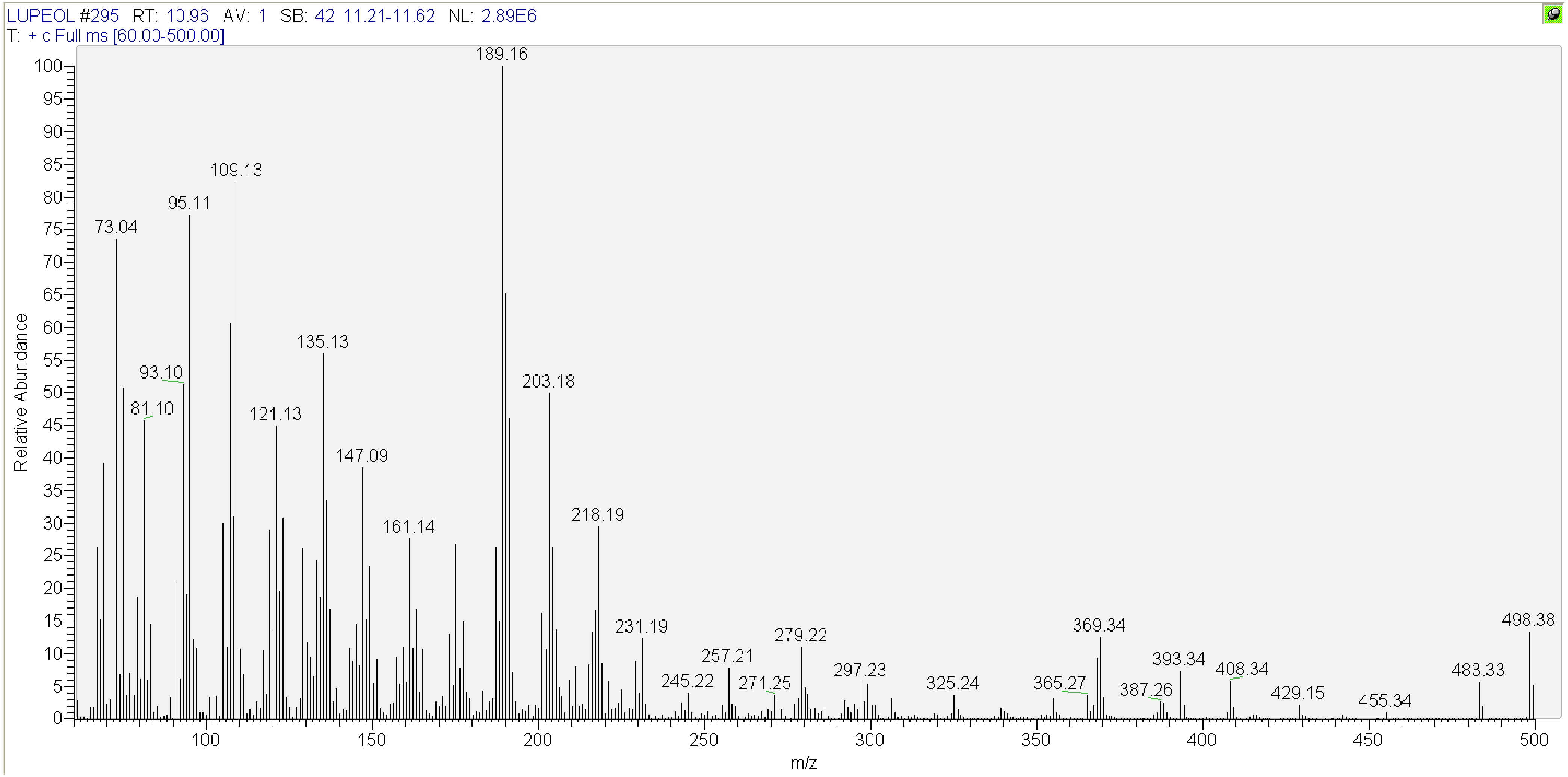

SF3-C: Ionization pattern of lupeol peak from AtLUS-6 transformant line

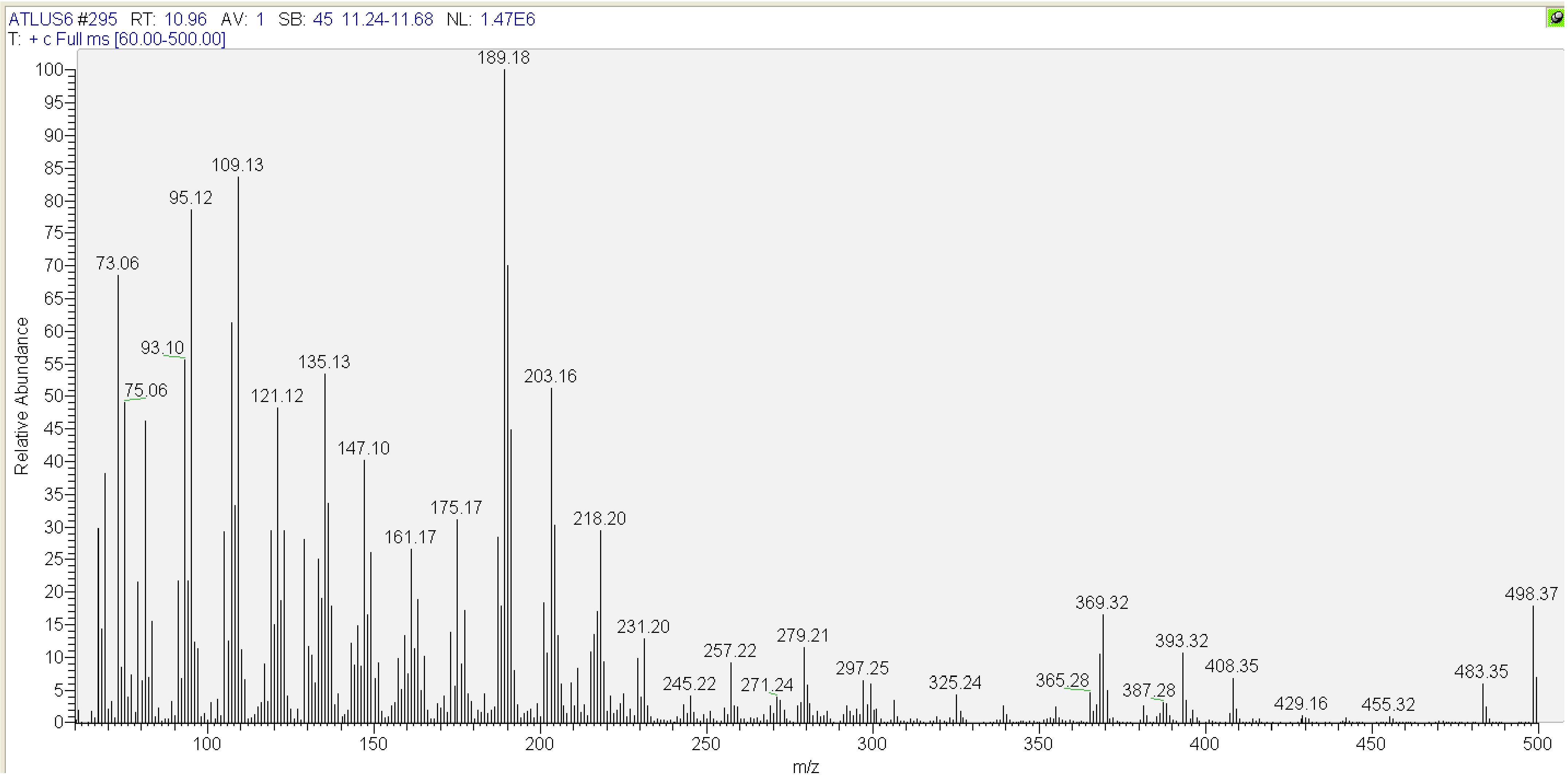

SF3-D: Ionization pattern of 2-3β hydroxylupane peak from AtLUS-6 transformant line

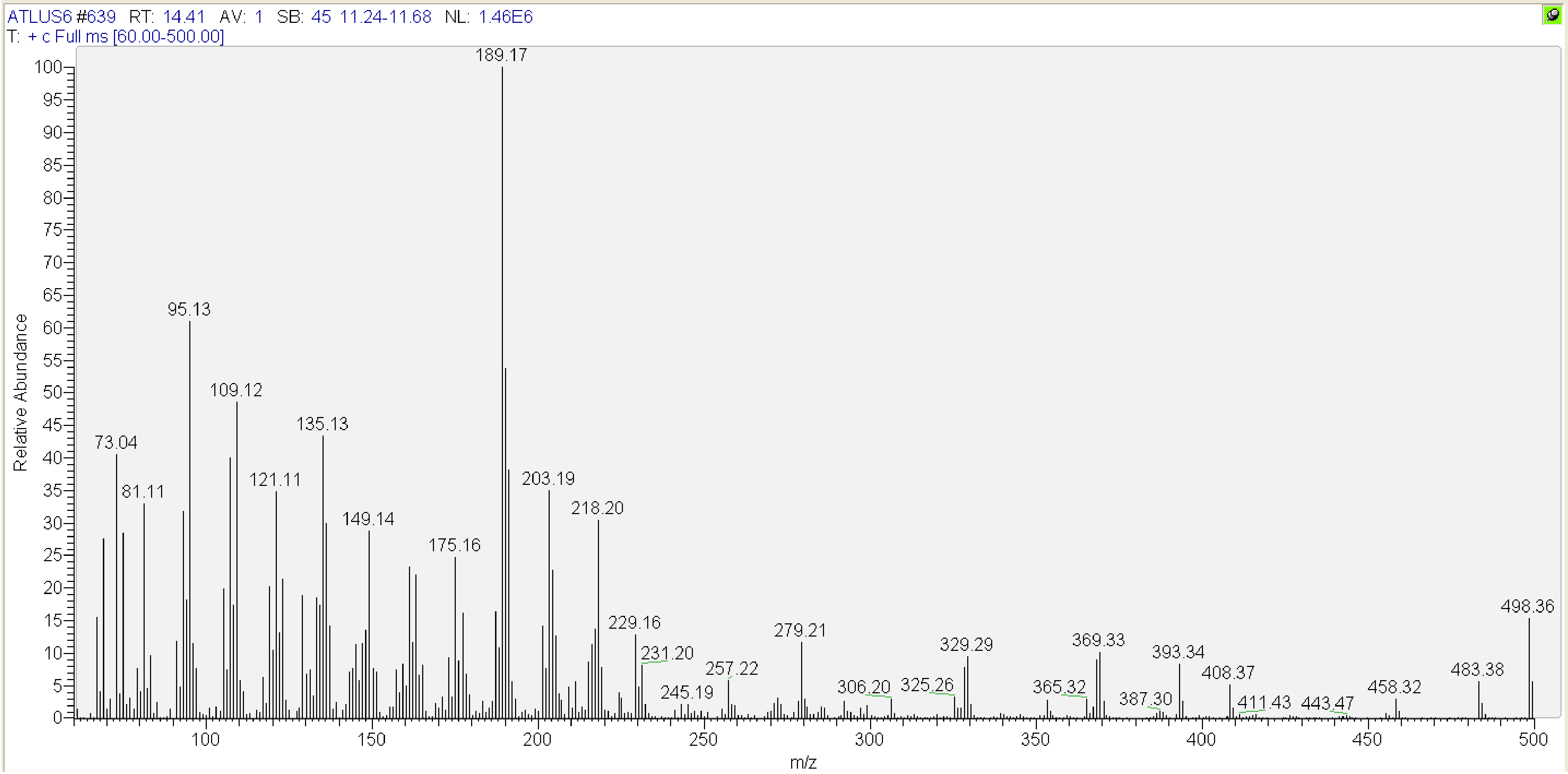

SF3-E: Ionization pattern of lupeol peak from LjLUS_MtCYP716A12_MtCPR_6 transformant line

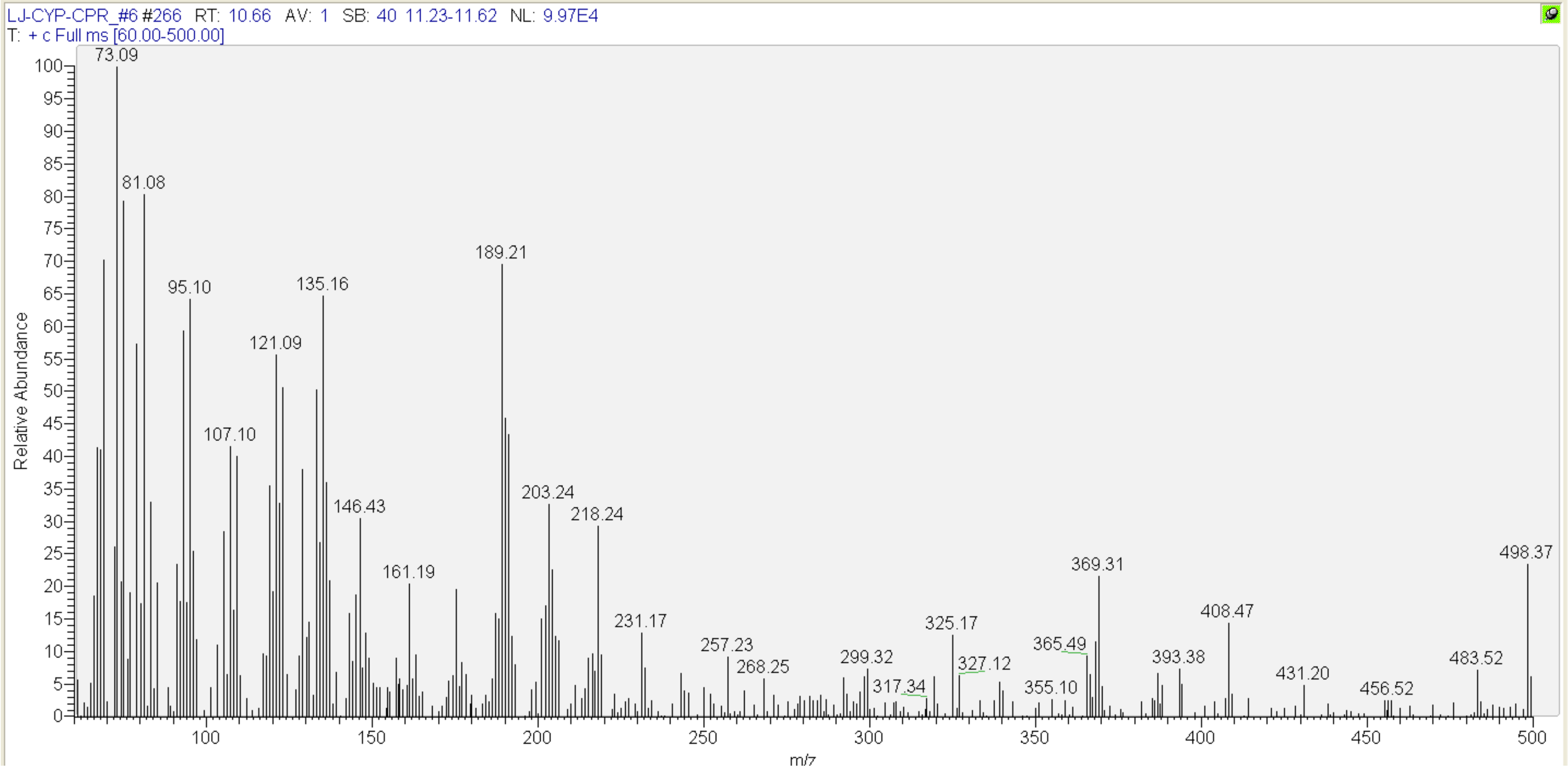

SF3-F: Ionization pattern of betulin peak from LjLUS_MtCYP716A12_MtCPR-6 transformant line

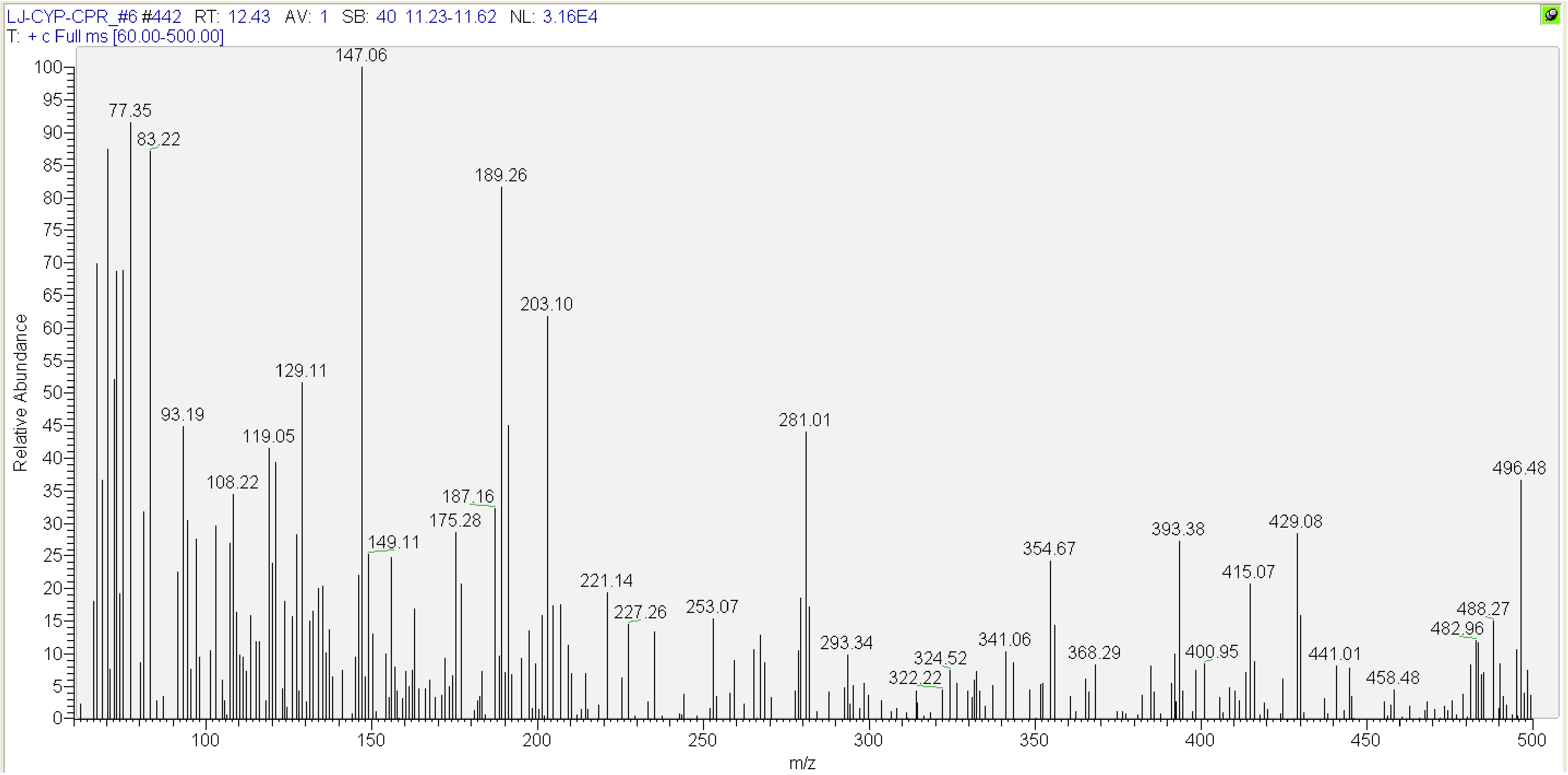

SF3-G: Ionization pattern of lupeol peak from LjLUS_MtCYP716A12λΔ71MtCPR_F28 transformant line

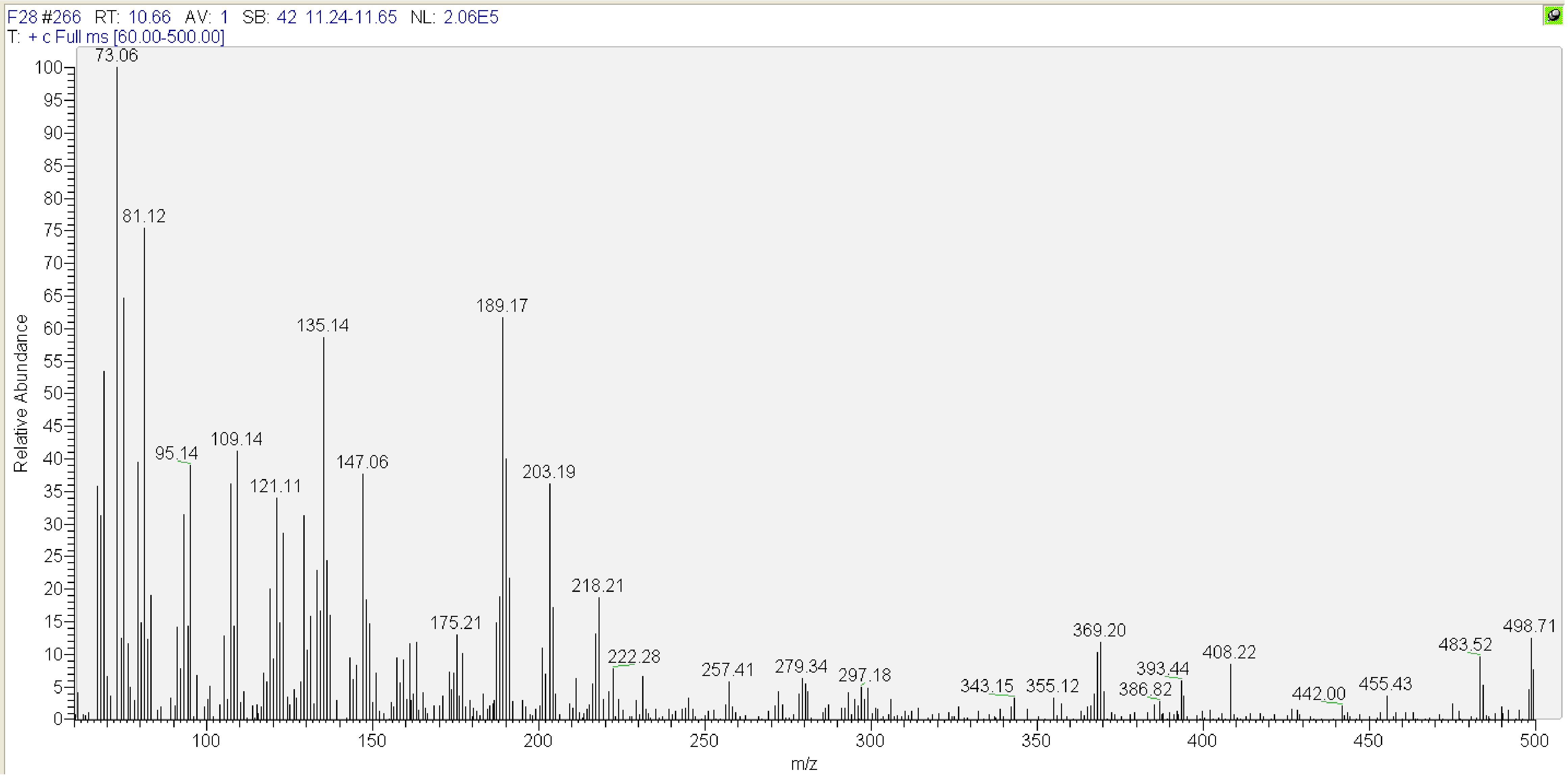

SF3-H: Ionization pattern of betulin peak from LjLUS_MtCYP716A12λΔ71MtCPR_F28 transformant line

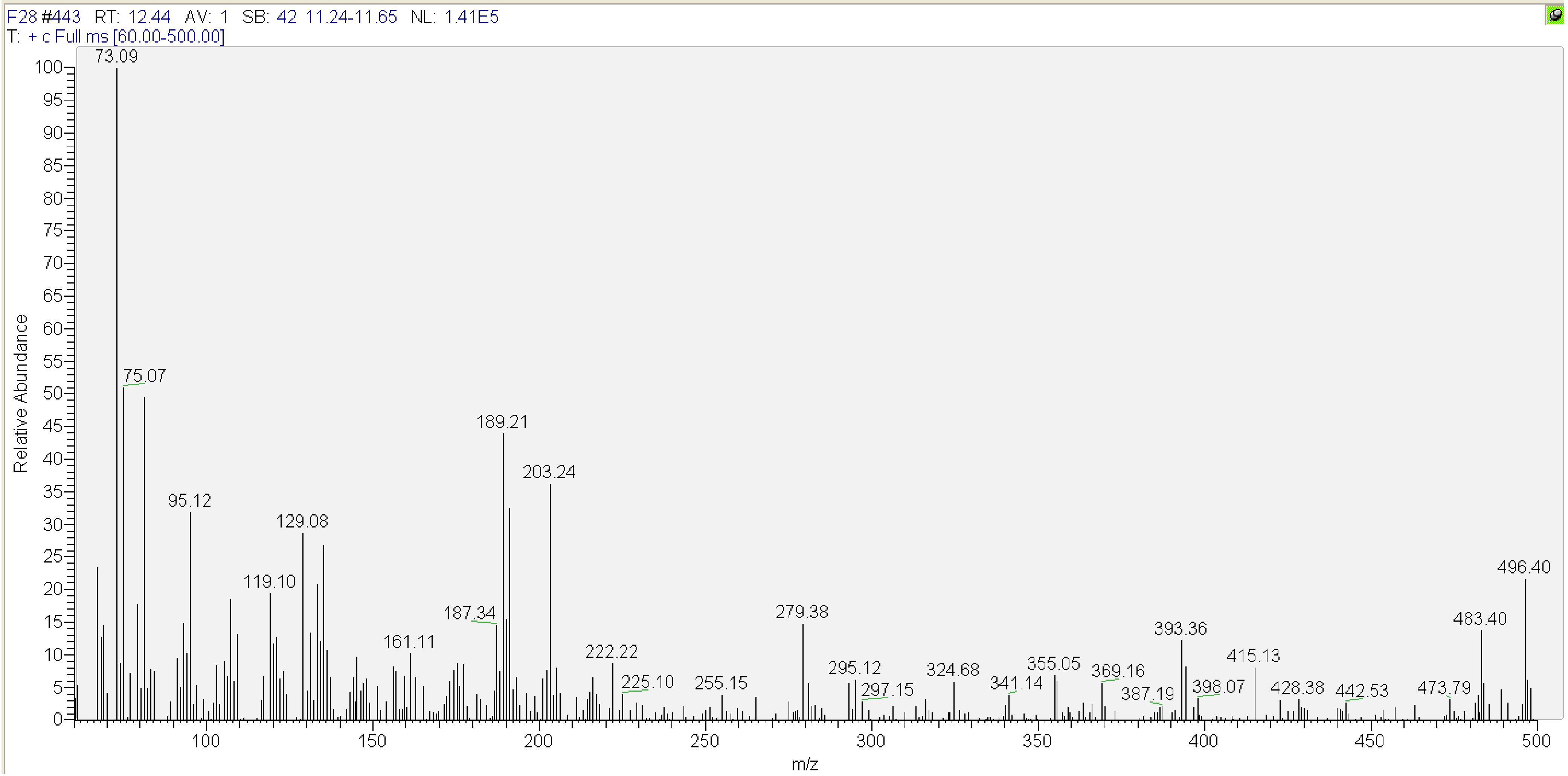

SF3-I: Ionization pattern of betulin peak from betulin standard

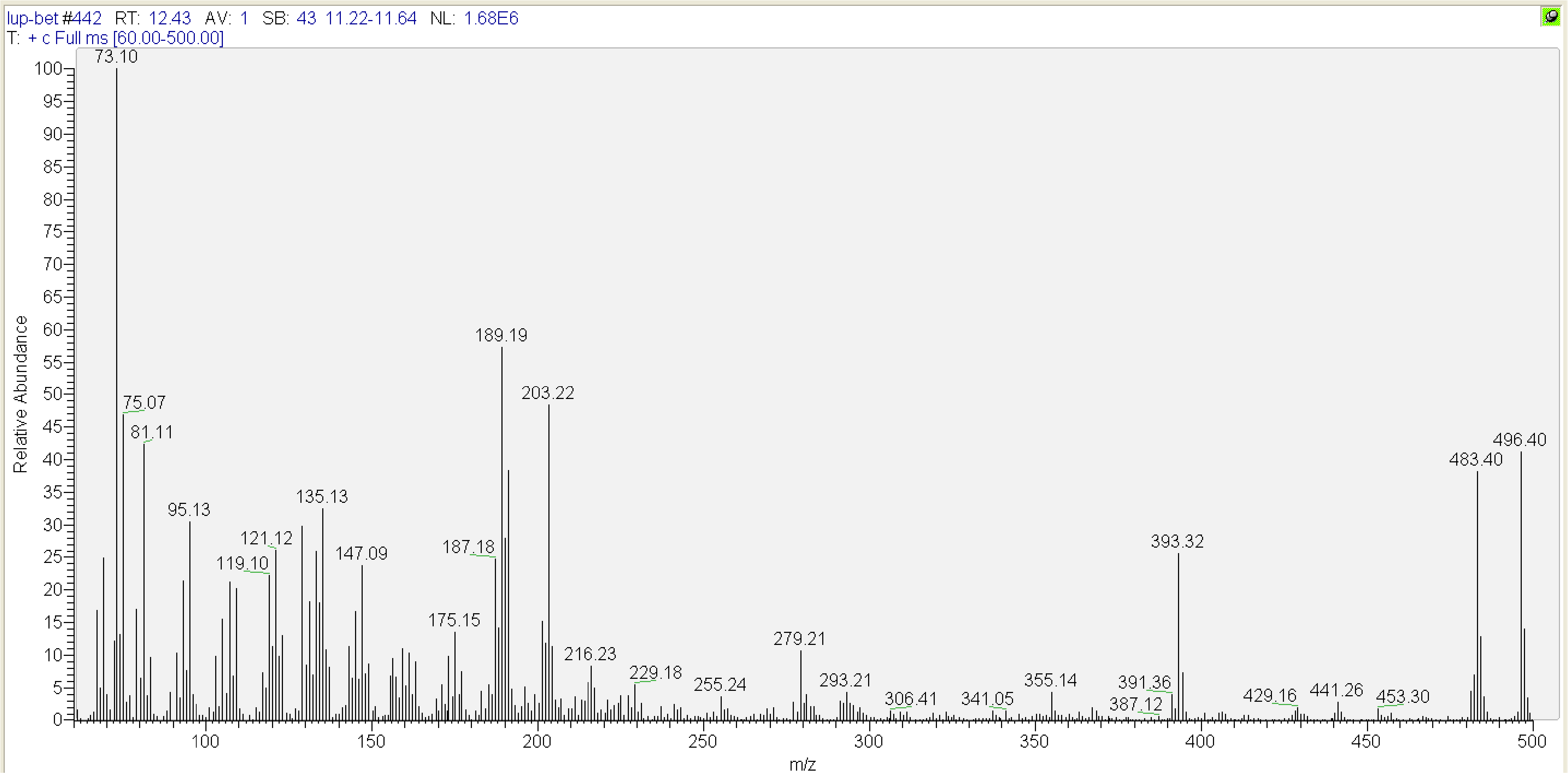

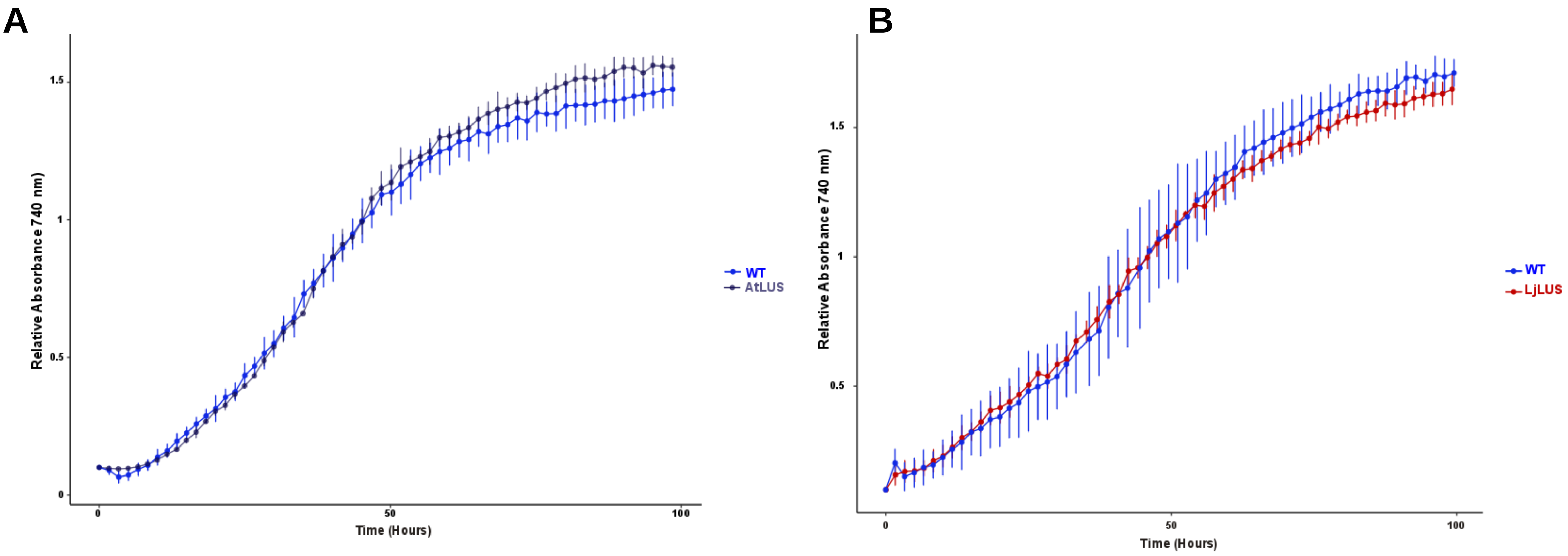

Supplemental Figure 4: Growth performance of the LUS transgenic lines in Algem® photobioreactors

A. Growth performance in Algem® photobioreactors of the AtLUS-6 line compared to the UTEX646 wild type strain (WT). **B.** Growth performance in Algem® photobioreactors of the LjLUS-25 strain compared to the CCAP1055/1 wild type strain (WT). Error bars represent standard deviation from three biological replicates.

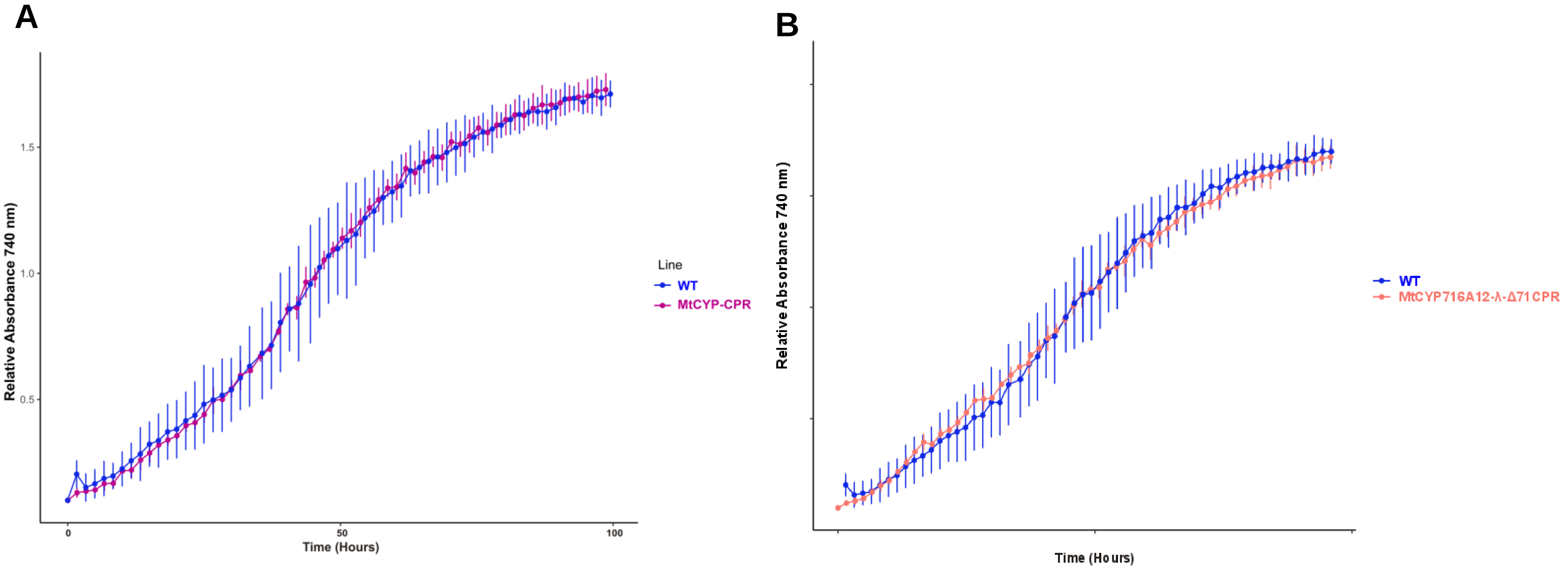

Supplemental Figure 5: Growth performance of the Betulin transgenic lines in Algem® photobioreactors
A. Growth performance of the selected MtCYP-MtCPR line transformed into the LjLUS-25 background. **B.** Growth performance of the MtCYP716A12-λ-Δ71CPR LjLUS co-transformation. Error bars represent standard deviation from three biological replicates.

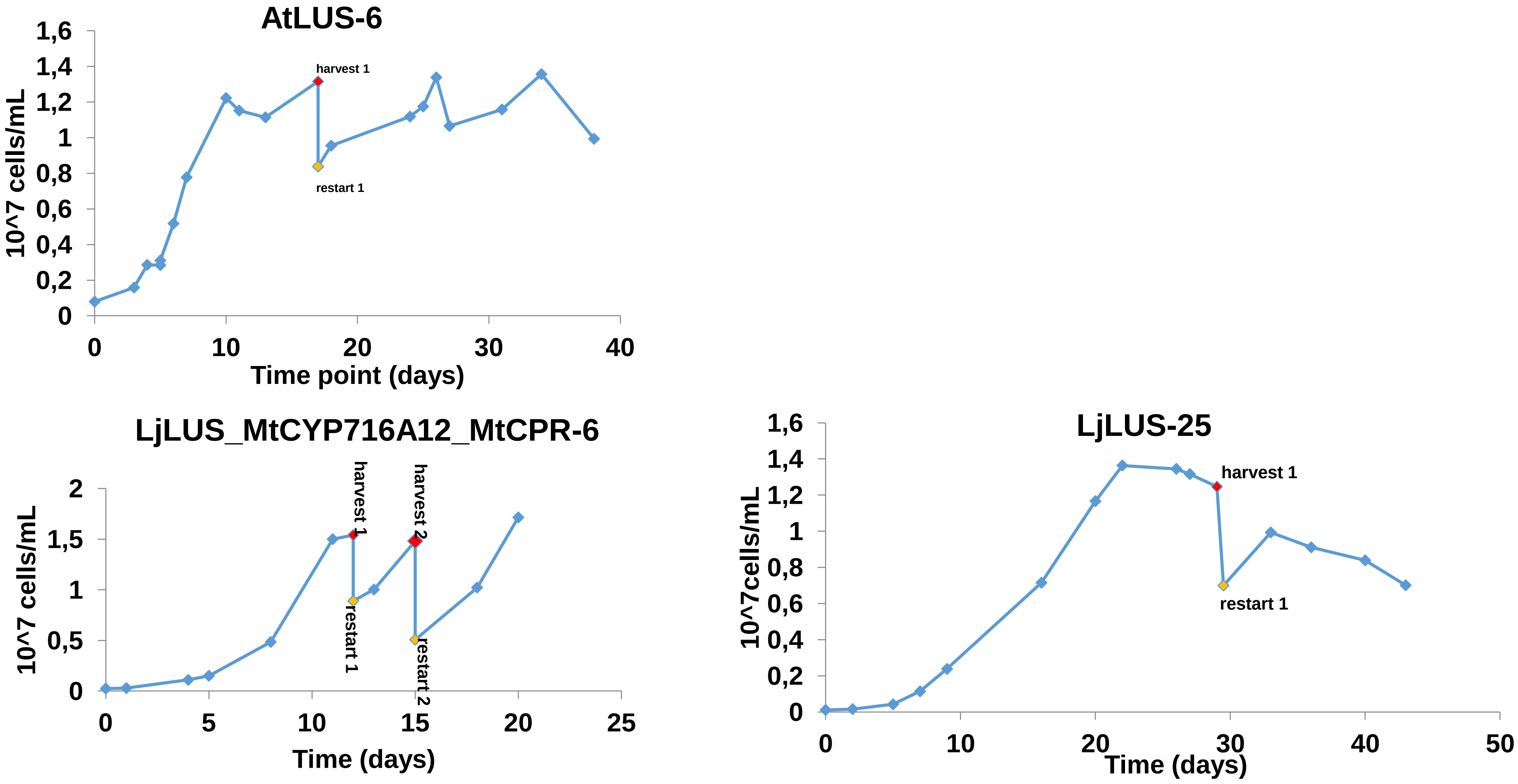

Supplemental Figure 6: Growth performance in 550L tubular PBR. Growth curves for the three lines grown in 550L tubular photobioreactor. Red dots indicate harvest time point, green dots the restart culture. For AtLUS-6 approximately 454L was harvested at day 17, and the same volume replenished with fresh F/2 medium into the PBR for a second harvest after 21 days. For LjLUS_25 285L have been harvested at day 29, then the culture started dying after restarting the PBR with fresh medium. For LjLUS_MtCYP716A12_MtCPR_6, 373L were first harvested after 12 days, and the same volume replenished with fresh F/2 medium into the PBR for a second harvest (455L) after 3 days.
