# Supplementary material for: Engineering the unicellular alga Phaeodactylum tricornutum for high‐value plant triterpenoid production
Source: Plant Biotechnol J. 2018 Jun 19;17(1):75–87. doi: 10.1111/pbi.12948 (PMC6330534; doi:10.1111/pbi.12948)
Supplement: Supplementary file 3 — Table S5 Repeated measures significance of the genes shown in Figure 6. [file PBI-17-75-s003.pdf]

# Table S5, Repeated measures significance of the genes shown in figure 6

R code used for the generation of the model:

```
repMes = function(exp, gene) {
  long = subset(exp, Target==gene)
  summary(aov(Expression ~ Sample*Day + Error(Flask), data=long))
}
```

Results of the repeated measures ANOVA:

## CAS

```
##
## Error: Flask
##        Df Sum Sq Mean Sq
## Sample  1 0.2549  0.2549
##
## Error: Within
##            Df Sum Sq Mean Sq F value  Pr(>F)
## Sample      1 0.0662  0.0662   0.999 0.33329
## Day         3 1.1227  0.3742   5.652 0.00853 **
## Sample:Day  3 0.3899  0.1300   1.963 0.16295
## Residuals  15 0.9932  0.0662
## ---
## Signif. codes:  0 '***' 0.001 '**' 0.01 '*' 0.05 '.' 0.1 ' ' 1
```

## Methylsterol Oxygenase

```
##
## Error: Flask
##        Df   Sum Sq  Mean Sq
## Sample  1 0.003447 0.003447
##
## Error: Within
##            Df Sum Sq Mean Sq F value  Pr(>F)
## Sample      1 0.3164  0.3164   4.021 0.06333 .
## Day         3 0.2834  0.0945   1.201 0.34338
## Sample:Day  3 1.3647  0.4549   5.781 0.00783 **
## Residuals  15 1.1804  0.0787
## ---
## Signif. codes:  0 '***' 0.001 '**' 0.01 '*' 0.05 '.' 0.1 ' ' 1
```

## Sterol Dehydrogenase

```
##
## Error: Flask
##        Df Sum Sq Mean Sq
## Sample  1  0.247   0.247
##
## Error: Within
##            Df Sum Sq Mean Sq F value  Pr(>F)
## Sample      1 0.7198  0.7198  10.678 0.00519 **
## Day         3 0.1457  0.0486   0.721 0.55509
## Sample:Day  3 0.4620  0.1540   2.284 0.12061
## Residuals  15 1.0112  0.0674
## ---
## Signif. codes:  0 '***' 0.001 '**' 0.01 '*' 0.05 '.' 0.1 ' ' 1
```

## HMGCoA-Synthase

```
##
## Error: Flask
##        Df    Sum Sq   Mean Sq
## Sample  1 7.387e-06 7.387e-06
##
## Error: Within
##            Df Sum Sq Mean Sq F value  Pr(>F)
## Sample      1 0.1757  0.1757   5.010 0.04079 *
## Day         3 0.9643  0.3214   9.167 0.00109 **
## Sample:Day  3 0.9018  0.3006   8.573 0.00149 **
## Residuals  15 0.5260  0.0351
## ---
## Signif. codes:  0 '***' 0.001 '**' 0.01 '*' 0.05 '.' 0.1 ' ' 1
```

## HMGR

```
##
## Error: Flask
##        Df Sum Sq Mean Sq
## Sample  1 0.7428  0.7428
##
## Error: Within
##            Df Sum Sq Mean Sq F value   Pr(>F)
## Sample      1 0.9322  0.9322  17.620 0.000777 ***
## Day         3 0.4647  0.1549   2.928 0.067884 .
## Sample:Day  3 0.6189  0.2063   3.899 0.030449 *
```

```
## Residuals  15 0.7936  0.0529
## ---
## Signif. codes:  0 '***' 0.001 '**' 0.01 '*' 0.05 '.' 0.1 ' ' 1
```

## IDI-SQS

```
##
## Error: Flask
##        Df Sum Sq Mean Sq
## Sample  1 0.5596  0.5596
##
## Error: Within
##            Df Sum Sq Mean Sq F value  Pr(>F)
## Sample      1 0.8175  0.8175  14.336 0.00179 **
## Day         3 0.1448  0.0483   0.847 0.48963
## Sample:Day  3 0.5466  0.1822   3.195 0.05402 .
## Residuals  15 0.8553  0.0570
## ---
## Signif. codes:  0 '***' 0.001 '**' 0.01 '*' 0.05 '.' 0.1 ' ' 1
```

## Monooxygenase

```
##
## Error: Flask
##        Df Sum Sq Mean Sq
## Sample  1 0.8848  0.8848
##
## Error: Within
##            Df Sum Sq Mean Sq F value   Pr(>F)
## Sample      1 1.0839  1.0839  19.033 0.000557 ***
## Day         3 0.4604  0.1535   2.695 0.083234 .
## Sample:Day  3 0.3599  0.1200   2.106 0.142310
## Residuals  15 0.8542  0.0569
## ---
## Signif. codes:  0 '***' 0.001 '**' 0.01 '*' 0.05 '.' 0.1 ' ' 1
```

## ISPD-MEP

```
##
## Error: Flask
##        Df  Sum Sq Mean Sq
## Sample  1 0.05656 0.05656
##
## Error: Within
##            Df Sum Sq Mean Sq F value  Pr(>F)
## Sample      1 0.2431  0.2431   2.969 0.10541
## Day         3 0.1976  0.0659   0.805 0.51064
## Sample:Day  3 1.8632  0.6211   7.587 0.00257 **
```

```
## Residuals  15 1.2280  0.0819
## ---
## Signif. codes:  0 '***' 0.001 '**' 0.01 '*' 0.05 '.' 0.1 ' ' 1
```

## IspE-MEP

```
##
## Error: Flask
##        Df Sum Sq Mean Sq
## Sample  1 0.1688  0.1688
##
## Error: Within
##            Df Sum Sq Mean Sq F value Pr(>F)
## Sample      1 0.0710 0.07101   1.416 0.2525
## Day         3 0.1332 0.04439   0.885 0.4710
## Sample:Day  3 0.5789 0.19297   3.849 0.0317 *
## Residuals  15 0.7521 0.05014
## ---
## Signif. codes:  0 '***' 0.001 '**' 0.01 '*' 0.05 '.' 0.1 ' ' 1
```
